# RET and PHOX2B Genetic Polymorphisms and Hirschsprung's Disease Susceptibility: A Meta-Analysis

**DOI:** 10.1371/journal.pone.0090091

**Published:** 2014-03-20

**Authors:** Chun-mei Liang, Dong-mei Ji, Xu Yuan, Ling-ling Ren, Juan Shen, Hai-yan Zhang

**Affiliations:** 1 Department of Hygiene Analysis and Detection, School of Public Health, Anhui Medical University, Hefei, People's Republic of China; 2 Department of Reproductive Medicine Center, The First Affiliated Hospital of Anhui Medical University, Hefei, People's Republic of China; 3 Department of Stomatology, The First Affiliated Hospital of Anhui Medical University, Hefei, People's Republic of China; University of Hong Kong, China

## Abstract

**Background:**

Many publications have evaluated the correlation between RET, PHOX2B polymorphisms and Hirschsprung's disease with conflicting results. We performed this meta-analysis to clarify the association of RET, PHOX2B polymorphisms with HSCR.

**Methods:**

We searched Pubmed, Elsevier Science Direct, China National Knowledge Infrastructure database, Chinese Biomedical database, Google scholar. The combined odds ratio (OR) with 95% CI was calculated to estimate the strength of the association. Heterogeneity and publication bias were also assessed.

**Results:**

In total, 16 studies concerning RET and 4 studies concerning PHOX2B were included in the meta-analysis. The effects of five polymorphisms of RET (rs1800858, rs1800860, rs1800861, rs10900297, rs2435357) and one polymorphism (rs28647582) of PHOX2B were evaluated. We found a significant correlation between RET polymorphisms and HSCR. For rs1800858, the overall ORs (95% CI) of the A versus G, AA versus GG, AA/AG versus GG and AA versus GG/AG were 3.81 (2.28–6.35); 8.36 (3.45–20.25); 3.59 (1.83–7.02); and 6.60 (3.66–11.89). For rs1800861, the comparison of subjects in the G versus T, GG versus TT, GG/TG versus TT and GG versus TT/TG were 2.85(1.81–4.47); 5.38(2.68–10.80); 3.07(2.17–4.34) and 4.14(1.84–9.30) respectively. For rs10900297, the comparison results showed statistically significant. (OR_C versus A_ = 5.05,95%CI = 4.16–6.13; OR_CC versus AA_ = 9.73, 95%CI = 5.94–15.94; OR_CC/AC versus AA_ = 5.31, 95%CI = 3.27–6.82; OR_CC versus AC/AA_ = 7.06,95%CI = 5.60–8.91.) But, for rs1800860, the GG/GA versus AA did not reach statistical association (OR = 3.77, 95% CI = 0.94–15.07) and the G versus A, GG versus AA, GG versus GA/AA were 2.23 (1.60–3.11);4.56 (1.14–18.27); 2.38 (1.66–3.43) respectively. For rs2435357, the T versus C, TT versus CC, TT/TC versus CC and TT versus CC/TC were 4.53 (3.27–6.27); 11.44 (5.67–23.10); 4.04 (2.92–5.57), and 9.01(5.25–15.46).The single polymorphism of PHOX2B gene wasn't related to the risk for HSCR.

**Conclusions:**

This meta-analysis shows a significant association between RET polymorphisms and HSCR.

## Introduction

Hirschsprung disease (HSCR) is a congenital malformation of the hindgut produced by a disruption in the neural crest cells (NCC) migration during embryonic development. This disorder results in an absence of intramural ganglion cells in the submucosal and myenteric plexuses producing a functional intestinal obstruction [1]. HSCR is classified, according to the extent of aganglionosis, into long-segment (L-HSCR, 20% of affected individuals) and short-segment (S-HSCR, 80%) forms, each with distinct genetic characteristics [2]. The incidence of this disease is generally 1 per 5000 of live births, with males about 3.5–7.8 times more likely to be affected than females [3] and it usually presents in infancy, although some patients present with persistent, severe constipation later in life. There are also differences among races, with a higher incidence in Asians at 2.8 per 10 000 of live births [4]. Besides, HSCR can be either familial or sporadic.

HSCR has a complex genetic etiology with several genes being described as associated with either isolated or syndromic forms, such as RET,EDNRB,GDNF,EDN3 and SOX10, NTN3, ECE1, PHOX2B [3]. RET encodes a receptor tyrosine kinase, which is expressed in cell lineages derived from the neural crest; plays a crucial role in the regulation of cell proliferation, migration, differentiation, and survival during embryogenesis; and functions as a receptor for growth factors of the glial cell line-derived neurotrophic factor (GDNF) family [5], [6]. There is growing evidence showing that some potentially functional single nucleotide polymorphisms (SNPs) of RET gene could act as low susceptibility factors and modify the phenotype of HSCR, especially in certain combinations of alleles, haplotypes [6]. The paired mesoderm homeobox 2b gene (PHOX2B) encodes a transcription factor (homeodomain protein) which is involved in the development of several noradrenergic neurone populations, besides, homozygous disruption of the PHOX2B gene results in absence of enteric ganglia, a feature which is reminiscent of HSCR [7] [8].

RET locates in 10q11.2 and is composed of 21 exons. Three common SNPs in the coding region of RET, c135G>A (rs1800858, A45A), c1296A>G (rs1800860, A432A) and c2307T>G (rs1800861, L769L) lie in exon2, exon7 and exon13 respectivly [5], [9], [10]. -1A>C (rs10900297) locates in the promoter region of RET [11]. Rs2435357 has been proven to lie in the enhancer-like sequence within intron 1 of the RET-protooncogene [3]. The PHOX2B maps to chromosome 4p12, encoding 314 homeodomain protein of amino acids. One common SNP, IVS2+100A>G (rs28647582) lies in intron 2 of PHOX2B [8]. Several case-control studies have investigated the association between these gene polymorphisms and Hirschsprung's disease risk, but the result is still not clear due to the inconsistence among those studies. The cause of this result may be due to sparseness of data, ethnic difference, different designs and publication bias. Meta-analysis has the advantage of reducing the risk of random error and obtaining a precise estimation for the major effect by combining data from all eligible studies [12]. Besides, to our knowledge, there were no quantitative reviews of the literature on the association between these gene polymorphisms and HSCR. Therefore, we conducted a meta-analysis of all available published case-control studies to verify the precise associations.

## Materials and Methods

### Identification and eligibility of relevant Studies

We conducted a comprehensive literature search in Pubmed, Elsevier Science Direct, China National Knowledge Infrastructure database, Chinese Biomedical database and Google scholar from January 2003 to December 2012 using the following search terms : hirschsprung disease, hirschsprung's disease; polymorphism, genetic; RET; and PHOX2B. Moreover, the references of the selected papers were also checked by hand-search for other potential articles that possibly have been missed in the initial search. Only papers in Chinese and English were included.

### Inclusion and exclusion criteria

The inclusion and exclusion criteria were drew up on the basis of the discussion studies. Studies eligible for this meta-analysis had to fulfill (1) the design type of study was a case-control study;(2) the study had examined the associations between the RET, PHOX2B gene polymorphisms and HSCR;(3) the frequencies of genotypes in case and control groups could be collected;(4) controls derived from a population within the same geographic area and ethnic background as HSCR cases. The exclusion criteria were as follows : (1) researches that did not meet the inclusion criteria; (2) the study reported useless or dupicated data.

### Data extraction

All of the data were extracted independently by two reviewers (Chun-mei Liang and Dong-mei Ji) according to the prespecified selection criteria. Potential disagreements were resolved by consensus. The following characteristics were extracted: name of first author, year of publication, racial ancestry of the study participants, genotypes and sample size, the polymorphisms investigated in the studies, the genotyping method, type of study.

### Statistical analyses

Allele frequencies of the RET and PHOX2B SNPs at genetic polymorphisms from the respective studies were determined by the allele counting method. All the statistical analyses were performed by Stata version 11.0 (Stata-Corp, College Station, TX).We assessed Hardy–Weinberg equilibrium (HWE) for the controls in each study by the Chi-square test. The odds ratio (OR) and its 95% confidence interval (95% CI) were estimated for each study by fixed or random effect model. Heterogeneity among studies was measured using the Chi-square based Q statistic [13]. We also quantified the effect of heterogeneity using I^2^ statistic which measures the severe degree of heterogeneity. I^2^ value ranges from 0 to 100% (I^2^ = 0–25%, no heterogeneity; I^2^ = 25–50%,moderate heterogeneity; I^2^ = 50–75%, large heterogeneity; I^2^ = 75–100%, extreme heterogeneity) [14]. If there was a statistical difference in terms of heterogeneity, a random effect model was selected to combine the data.Otherwise, a fixed effect model was used. Visual inspection of asymmetry in funnel plots was conducted. Begger's rank correlation method was used to statistically assess the publication bias (*P*<0.05 was considered to be representive of statistically significant publication bias).

### Main results of meta-analysis

**Figure 1:**
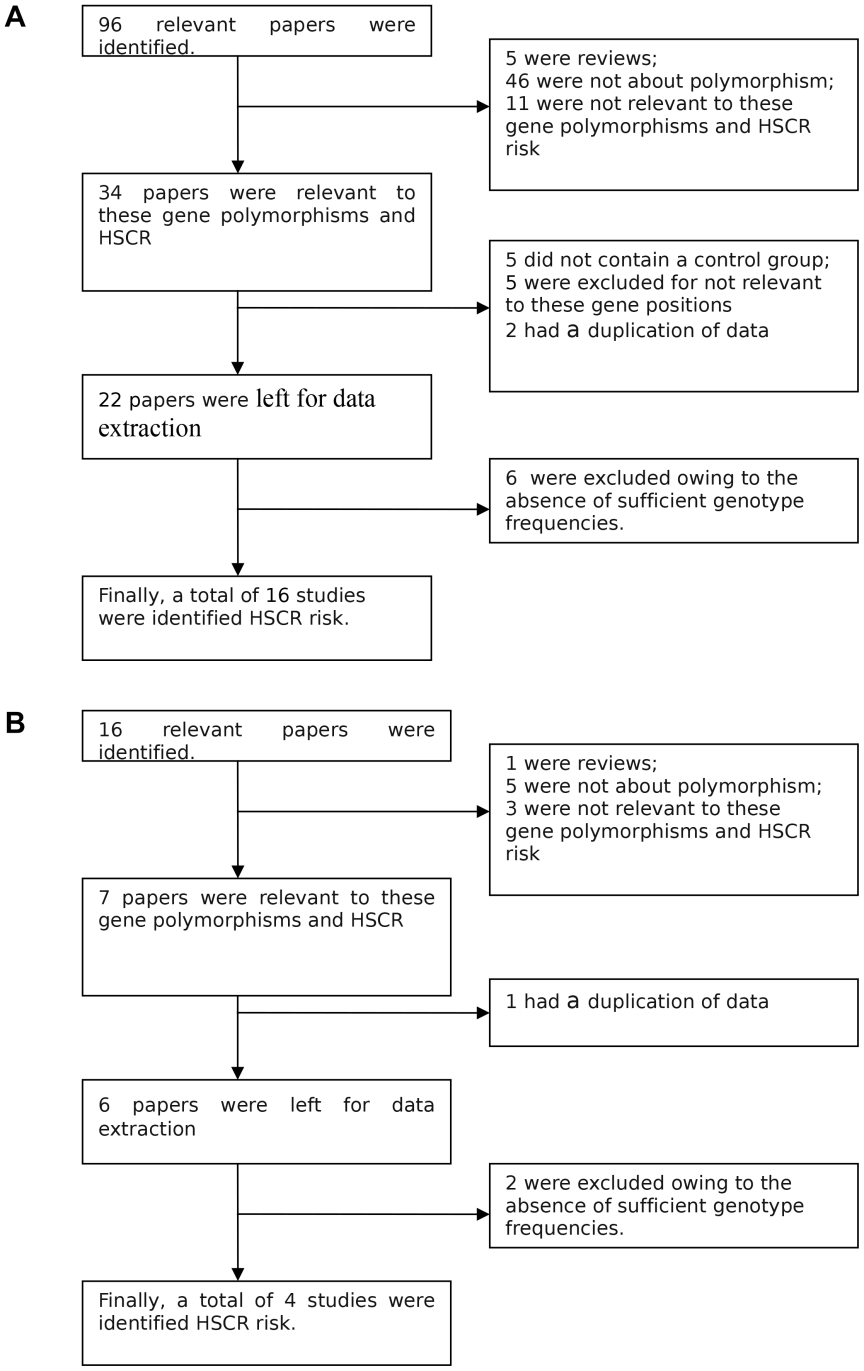
Process of selecting studies about RET and PHOX2B gene.

The process of selecting studies was showed in Figure 1. For RET gene, a total of 96 papers were identified after an initial search,11 of which were published in Chinese. Based on the exclusion and inclusion criteria in original manuscript, after reading the titles or abstracts, 11 papers were excluded for not relevant to these gene polymorphisms with HSCR risks; 5 were reviews; 46 were not about polymorphisms. After reading full texts of the remaining 34 papers, 5 did not contain a control group; 5 were excluded for not relevant to the loci;2 had a duplication of data. Thus, 22 articles were left for data extraction. Of these, 6 studies were excluded owing to the absence of sufficient genotype frequencies.Finally, 16 separate studies including 1,527 cases and 1,963 controls were considered in the current meta-analysis. Among these studies, there were five SNPs discussed in this meta-analysis (rs1800858, rs1800860, rs1800861, rs10900297, rs2435357). The controls of studies [15]–[17] for rs1800858 and the controls of studies [15] for rs1800861 were not conformed to HWE (Table 1). The characteristics of the studies that investigated the association of RET gene polymorphisms with HSCR were showed in Table 1. For PHOX2B gene, a total of 16 papers were identified after an initial search, 3 were published in Chinese.Based on the exclusion and inclusion criteria mentioned above, 3 papers were excluded for the irrelevance of the gene polymorphisms with HSCR risks; 1 was review; 5 were not about polymorphisms, 1 had a duplication of data. Then, 6 articles were left for data extraction. Of these, 2 studies were excluded owing to the absence of sufficient genotype frequencies. Finally, 4 separate studies including 372 cases and 511 controls were considered in this meta-analysis. The characteristics of the four studies were showed in Table 2. The controls of studies [8] for rs28647582 was not conformed to HWE (Table 2).

**Table 1:** Characteristics of studies included in this meta-analysis about RET gene.

First author	Year	Population	Race	Cases/controls	Geme polymorphism	Genotype method	Type of study	HWE
Phusantisampan	2012	Thai	Asia	68/120	rs1800858(G>A)	PCR–RFLP	Hospital-based	0.12
					rs1800861(T>G)	TaqMan		0.77
					rs2435357(T>C)	TaqMan		0.40
Liu	2010	Chinese	Asia	125/148	rs1800858(G>A)	PCR	Hospital-based	0.79
					rs1800860(A>G)			0.86
Tou	2011	Chinese	Asia	123/168	rs1800858(G>A)	PCR	Hospital-based	0.72
					rs1800860(A>G)			0.81
					rs1800861(T>G)			0.09
					rs10900297(A>C)			0.77
Miao	2010	Chinese	Asia	315/352	rs10900297(A>C)	PCR	Hospital-based	0.99
					rs2435357(T>C)		Hospital-based	0.39
Fitze	2003	German	Europe	80/120	rs1800858(G>A)	NA	Hospital-based	0.90
					rs10900297(A>C)			0.97
Burzynski	2004	Netherlander	Europe	105/126	rs1800858(G>A)	NA	Hospital-based	0.24
Sadewa	2008	Indonesians	Asia	34/46	rs1800861(T>G)	PCR-RFLP	Hospital-based	0.06
Garcia-Barcelo	2005	Chinese	Asia	172/194	rs1800858(G>A)			0.54
					rs1800861(T>G)			0.08
					rs10900297(A>C)			0.36
Li	2011	Chinese	Asia	80/80	rs1800860(A>G)	PCR	Hospital-based	0.29
					rs18008611(T>G)			0.12
Du	2006	Chinese	Asia	94/122	rs1800858(G>A)	PCR	Hospital-based	<0.01
					rs1800861(T>G)			<0.01
Zhang	2005	Chinese	Asia	16/40	rs1800858(G>A)	PCR	Hospital-based	<0.05
Zhao	2012	Chinese	Asia	80/80	rs1800858(G>A)	PCR-HRM	Hospital-based	<0.01
Wang	2006	Chinese	Asia	52/120	rs10900297(A>C)	PCR	Hospital-based	0.41
Arnold	2008	Caucasian	Europe	62/30	rs2435357(T>C)	TaqMan	Hospital-based	0.66
Pini Prato	2009	Italian	Europe	22/85	rs2435357(T>C)	PCR	Hospital-based	0.44
Zhang	2007	Chinese	Asia	99/132	rs2435357(T>C)	PCR	Hospital-based	0.54

**Table 2:** Characteristics of studies included in this meta-analysis about PHOX2B gene.

First author	Year	Population	Race	Cases/controls	Geme polymorphism	Genotype method	Type of study	HWE
Garcia-Barcelo	2003	Chinese	Asia	91/71	rs28647582(A>G)	PCR	Hospital-based	0.07
Liu	2009	Chinese	Asia	100/96	rs28647582(A>G)	PCR	Hospital-based	<0.05
Dou	2007	Chinese	Asia	123/194	rs28647582(A>G)	PCR	Hospital-based	0.09
Xiao	2009	Chinese	Asia	58/150	rs28647582(A>G)	PCR	Hospital-based	0.54

### rs 28647582 polymorphism

Totally, there were 4 studies [7], [8], [18], [19] including 372 cases and 511 controls inspecting the correlation between rs 28647582 and HSCR risk. The comparisons of all genotypes did not detect any statistical association. The results were showed in Table 3 and supplement S2.

**Table 3:** Main results of the meta-analysis.

Gene polymorphism	Number of studies		Test of association			Test of heterogeneity			Publication bias
		Comparison	OR	95%CI	*P* value	Q	*P* value	I^2^(%)	*P* value (Begg's)
rs28647582	4	GG vs AA	2.05	0.94–4.48	0.07	3.63	0.305	17.3	0.497
	4	GG vs GA+AA	2.14	0.98–4.69	0.06	3.12	0.373	4.0	1.000
	4	GG+GA vs AA	0.76	0.40–1.45	0.41	12.69	0.005	76.4	0.174
	4	G vs A	0.86	0.46–1.60	0.64	14.90	0.002	79.9	0.174
rs1800858	9	AA vs GG	8.36	3.45–20.25	0.000	68.06	0.000	88.2	0.835
	9	AA+GA vs GG	3.59	1.83–7.02	0.000	55.29	0.000	85.5	0.835
	9	AA vs GA+GG	6.60	3.66–11.89	0.000	53.05	0.000	84.9	0.835
	9	A vs G	3.81	2.28–6.35	0.000	99.13	0.000	91.9	1.000
rs1800860	2	GG vs AA	4.56	1.14–18.27	0.032	0.89	0.347	0.0	0.317
	3	GG vs GA+AA	2.38	1.66–3.43	0.000	0.64	0.728	0.0	0.602
	2	GG+GA vs AA	3.77	0.94–15.07	0.061	0.87	0.351	0.0	0.317
	3	G vs A	2.23	1.60–3.11	0.000	0.80	0.669	0.0	0.117
rs1800861	6	GG vs TT	5.38	2.68–10.80	0.000	16.29	0.006	69.3	0.851
	6	GG+TG vs TT	3.07	2.17–4.34	0.000	5.23	0.388	4.5	0.348
	6	GG vs TT+TG	4.14	1.84–9.30	0.001	50.09	0.000	90.0	0.348
	6	G vs T	2.85	1.81–4.47	0.000	30.02	0.000	83.3	0.188
rs10900297	5	CC vs AA	9.73	5.94–15.94	0.000	1.45	0.835	0.0	1.000
	5	CC+AC vs AA	5.31	3.27–8.62	0.000	1.43	0.839	0.0	0.624
	5	CC vs AC+AA	7.06	5.60–8.91	0.000	4.84	0.304	17.3	1.000
	5	C vs A	5.05	4.16–6.13	0.000	4.02	0.403	0.5	0.624
rs2435357	5	TT vs CC	11.44	5.67–23.10	0.000	10.06	0.039	60.3	0.327
	5	TT+TC vs CC	4.04	2.92–5.57	0.000	4.39	0.355	9.0	0.624
	5	TT vs TC+CC	9.01	5.25–15.46	0.000	10.30	0.036	61.2	0.327
	5	T vs C	4.53	3.27–6.27	0.000	9.73	0.045	58.9	1.000

### rs1800858 polymorphism

In total, we identified nine studies [3], [15]–[17], [20]–[24] including 863 cases and 1,118 controls examining the relation between rs1800858 and HSCR risk. We found a prominent association of rs1800858 gene polymorphism with HSCR. The results were presented in Table 3 and supplement S2.

### rs1800860 polymorphism

On the basis of three studies [20], [21], [25] involving 328 cases and 396 controls, an association was not observed only in the GG/GA versus AA genotype. The detailed results were listed in Table 3 and supplement S2.

### rs1800861 polymorphism

Six studies [3], [15], [21], [24]–[26]including 571 cases and 730 controls researched the role of rs1800861 polymorphism in the occurrence of HSCR. The comparisons of all genotypes were detected for statistical associations. The results were listed in Table 3 and supplement S2.

### rs10900297polymorphism

Totally, five studies [21], [22], [24], [27], [28]which contained 742 cases and 954 controls investigated the possible effect of the rs10900297 polymorphism on the development of HSCR. We also successfully obtained a significant association of rs10900297 gene polymorphism with HSCR. The results were showed in Table 2 and supplement S2.

### rs2435357 polymorphism

In all, five studies [3], [27], [29]–[31] including 566 cases and 719 controls assessed the association between rs2435357 polymorphism and HSCR. Amongst them, two studies [3], [29] were conducted in European population. We found a very significant association of rs2435357 gene polymorphism with HSCR. The detailed results were showed in Table 3 and supplement S2.

### Subgroup analyses

Moreover, we performed subgroup analyses by the race. The detailed results were showed in Table 4.Seven studies [3], [15]–[17], [20], [21], [24] were conducted in Asia and Two [22], [23] were in Europe for rs1800858 polymorphism,. When stratified by race, the results remained statistical significant. For other SNPs, we were not able to stratify with insufficient information of subgroup.

**Table 4:** The results of subgroup analyses.

Gene polymorphism	Comparison	OR (95%CI)	
		Asia	Europe
rs1800858	AA vs GG	5.92 (2.14–16.34)	26.71 (13.92–51.24)
	AA vs AG+GG	5.28 (2.66–10.46)	14.78 (8.34–26.16)
	AA+AG vs GG	2.89 (1.29–6.48)	7.04 (4.41–11.24)
	A vs G	3.25 (1.75–6.04)	6.52 (4.83–8.81)

### Sensitivity Analysis

Although the distribution of genotypes in the controls in some studies did not follow HWE, the corresponding pooled OR and between-study heterogeneity were not significant altered without these studies for rs1800858. (AA vs. GG: OR = 8.56, 95% CI = 2.47–29.71,P_heterogeneity_ = 0.000; AA vs. AG/GG: OR = 5.91,95% CI = 2.55–13.69, P_heterogeneity_ = 0.000; AA/AG vs. GG: OR = 3.58,95% CI = 1.46–8.81, P_heterogeneity_ = 0.000; A vs. G: OR = 3.46, 95% CI = 1.73–6.95, P_heterogeneity_ = 0.000). However,sensitivity analysis showed that the studies by Du et al was the main origin of heterogeneity for rs1800858. The heterogeneity significantly decreased when this study was excluded(P_heterogeneity_ = 0.10 for GG vs. TT), while the value of pooled OR was not significantly altered without this studies (GG vs. TT:OR = 7.41, 95% CI = 4.89–11.23).(supplement S4)

### Publication bias

Both Begg's funnel plot and Egger's test were performed to assess the publication bias of the studies. The shape of funnel plots of all contrasts models was summetrical, and *P* values of Egger's tests were more than 0.05,providing statistical evidences of funnel plots'summetry.The results of Egger's test suggested no publication bias (Table 3 and supplement S3).

## Discussion

Hirschsprung disease (HSCR), a congenital malformation characterized by intestinal obstruction and colonic distension in newborns, and constipation in adults, that occurs in 1 in 5000 live births [3]. Mutations of the RET proto-oncogene have been detected in HSCR cases, nucleotide changes include microdeletions, insertions, variants affecting the correct RNA splicing, nonsense mutations, silent mutations, and missense mutations, with more than 100 different mutations described so far [32]. However, RET mutations have been detected in only up to 50% of familial patients and in 7%–35% of sporadic cases [33]. Recently, SNP, as the third generation of genetic markers, becomes the focus study. Studies showed that the SNPs of multiple introns and exons of RET expressed very highly or very lowly, which was associated with the phenotype of HSCR [16]. Besides, the SNPs of other genes such as PHOX2B also have been reported to relate to HSCR.

Although the association between polymorphisms of RET, PHOX2B and HSCR risk has been reported by a number of studies, the conclusions remained controversial due to the inconsistent findings. For rs1800858 polymorphism, Phusantisampan et al [3] suggested the A allele was protective against HSCR and G was risk allele, however, other studies [15]–[17], [20]–[24] found that the carriers of the G allele showed significantly increased risk of HSCR. Liu et al [20] found that rs1800860 played a protective role in the pathogenesis of HSCR in Chinese population, which differed from the findings of Tou et al [21], Li et al [25] and Lantieri et al [28]. Sadewa et al [26] showed the (GG) genotype of rs1800861gene did not associate with the risk of HSCR occurance., but some studies [3], [21], [24] suggested that the (GG) genotype was was significantly higher in HSCR patients compared to healthy controls. Liu et al [8] and Xiao et al [19] suggested that the (GG) genotype of rs28647582 was a risk genotype in HSCR patients, but Dou et al [18] showed that the (GG) genotype of rs28647582 gene did not associate with the risk of HSCR occurance. Meta-analysis is a powerful method for quantitatively summarizing the results from different studies, so we conducted this study to obtain a more comprehensive and reliable conclusion. In brief, our study included five gene polymorphisms of RET (rs1800858,rs1800860, rs1800861, rs10900297 and rs2435357) and one gene polymorphism (rs28647582) of PHOX2B.For all we know, this is the first meta-analysis investigating the association between these gene polymorphisms and HSCR.

Because the sample size was larger than previous ones, the meta-analysis reduced the probability that random error produced false-positive or false-negative association. We found a significant association between these gene polymorphisms (rs1800858,rs1800861,rs10900297,rs2435357) and HSCR demonstrating that the haplotype composited with more HSCR-risk alleles rendered the hosts more susceptible to HSCR. We failed to find an association in GG/GA versus AA genotype for rs1800860 polymorphism(OR_GG/GA vs. AA_ = 3.77, 95% CI = 0.97–15.04, *P*_OR_ = 0.062), but comparisons of the remaining combinations were statistically significant. The reason there was no association between them may be because only two original studies were included into the meta-analysis. For this reason, more original studies regarding the association between rs1800860 gene polymorphism and HSCR are necessary for accurate results. In addition, we found no difference between cases and controls in the comparisons of all genotypes of rs28647582 in this meta-analysis, which indicated that the polymorphism of rs28647582 may not associate with the susceptibility of HSCR.

Subgroup analyses by ethnicity further identified the significant association between rs1800858 gene polymorphisms and HSCR. This demonstrated that the polymorphic variance of this gene did not exist between European and Asian population, although the incidence of this congenital megacolon varied from 1.5 in Caucasians to 2.8 in Asians for each 10,000 newborns [4].

It should be noted that there were some limitations in this study. First, as the relevant investigations were not well-established, our sample size of this study was not adequate. Second, because only published studies were included in this study, publication bias may have occurred, even though no statistical test bias was found. Third, significant heterogeneity was observed among our comparison, especially for rs1800858 and rs1800861. Many aspects may cause heterogeneity, for example, the differences of experimental methods and the source of cases and controls in different studies and the clinical classification of HSCR. It was not a major problem because HSCR itself patients population may contribute to the heterogeneity. Fourth, because we could not get enough information from these studies, the meta-analysis did not conduct a subgroup analysis about concerning familial and sporadic HSCR. Fifth, the other diseases such as MEN2A, MEN2B,FMTC,MTC also have the RET mutations. The prevalence of Hirschsprung disease in multiple endocrine neoplasia type 2 cases was recently determined to be 7.5% and the co-occurrence of Hirschsprung disease and multiple endocrine neoplasia type 2 has been reported in at least 22 families so far [34]. We can not include these data in the analysis of the current paper in a combined and/or separated cohort with cases with HSCR only because we can not obtain the concrete data of those papers having been published about this aspect. Sixth, there are 2 single nucleotide polymorphisms (SNPs) in this enhancer sequence, rs2435357(Enh1∶C>T) (also called RET+3) and rs2506004 (Enh2∶C>A) (also called IVS+9494),of which the Enh1-T and the Enh2-A alleles both are strongly associated with HSCR [35] [36]. We can not include the data about rs2506004 in the analysis of the current paper because we can not obtain the concrete data of those papers having been published about this aspect. Finally, the language of the published studies included in our meta-analysis was limited in English and Chinese and, the publication bias may occur.

In summary, findings from this meta-analysis indicate that the SNPs of (rs1800858, rs1800860, rs10900297 and rs2435357) polymorphisms is significantly associated with an increased risk of HSCR, and the SNPs of rs28647582 may not relate to the susceptibility of HSCR. More work is needed to further investigate the association of the other RET SNPs with HSCR. Besides, future studies are recommended to identify the possible genetic interactions in this association.

## Supporting Information

Supplement S1 **PRISMA 2009 Checklist.**
(DOC)

Supplement S2 **Forest plots about RET,PHOX2B gene polymorphisms and HSCR.**
(DOC)

Supplement S3 **Funnel plots about RET,PHOX2B gene.**
(DOC)

Supplement S4 **Sensitivity analysis after exclusion of three studies deviating from HWE on the association of rs1800858 and rs1800861 gene polymorphisms with HSCR risk.**
(DOC)
