# Supplementary material for: RET and PHOX2B Genetic Polymorphisms and Hirschsprung's Disease Susceptibility: A Meta-Analysis
Source: PLoS One. 2014 Mar 20;9(3):e90091. doi: 10.1371/journal.pone.0090091 (PMC3961244; doi:10.1371/journal.pone.0090091)
Supplement: Supplement S2 — Forest plots about RET,PHOX2B gene polymorphisms and HSCR. (DOC) [file pone.0090091.s002.doc]

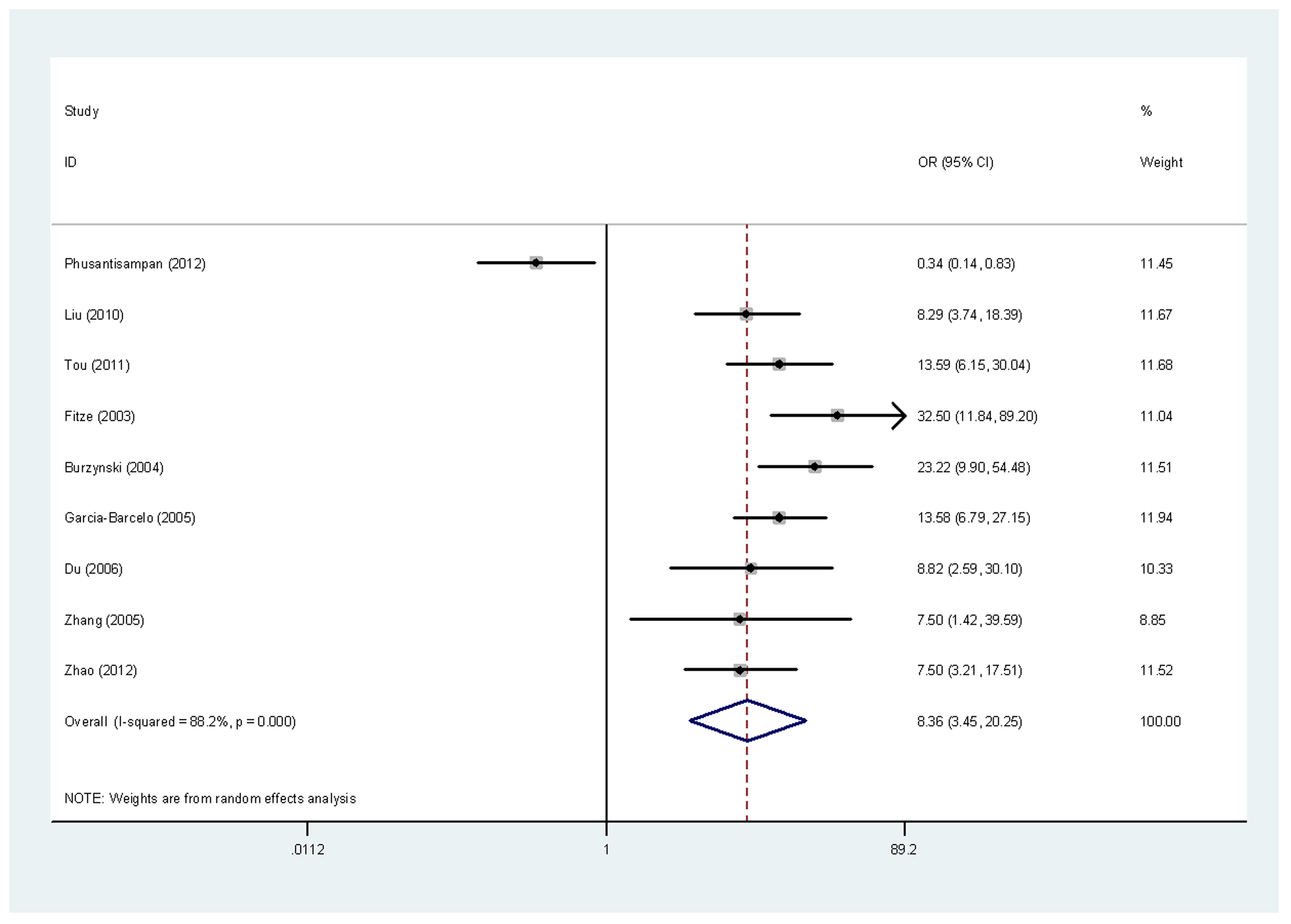


**Figure 1. Meta-analysis with a radom-effect model for the ORs of HSCR risk associated with rs1800858 AA vs. GG**


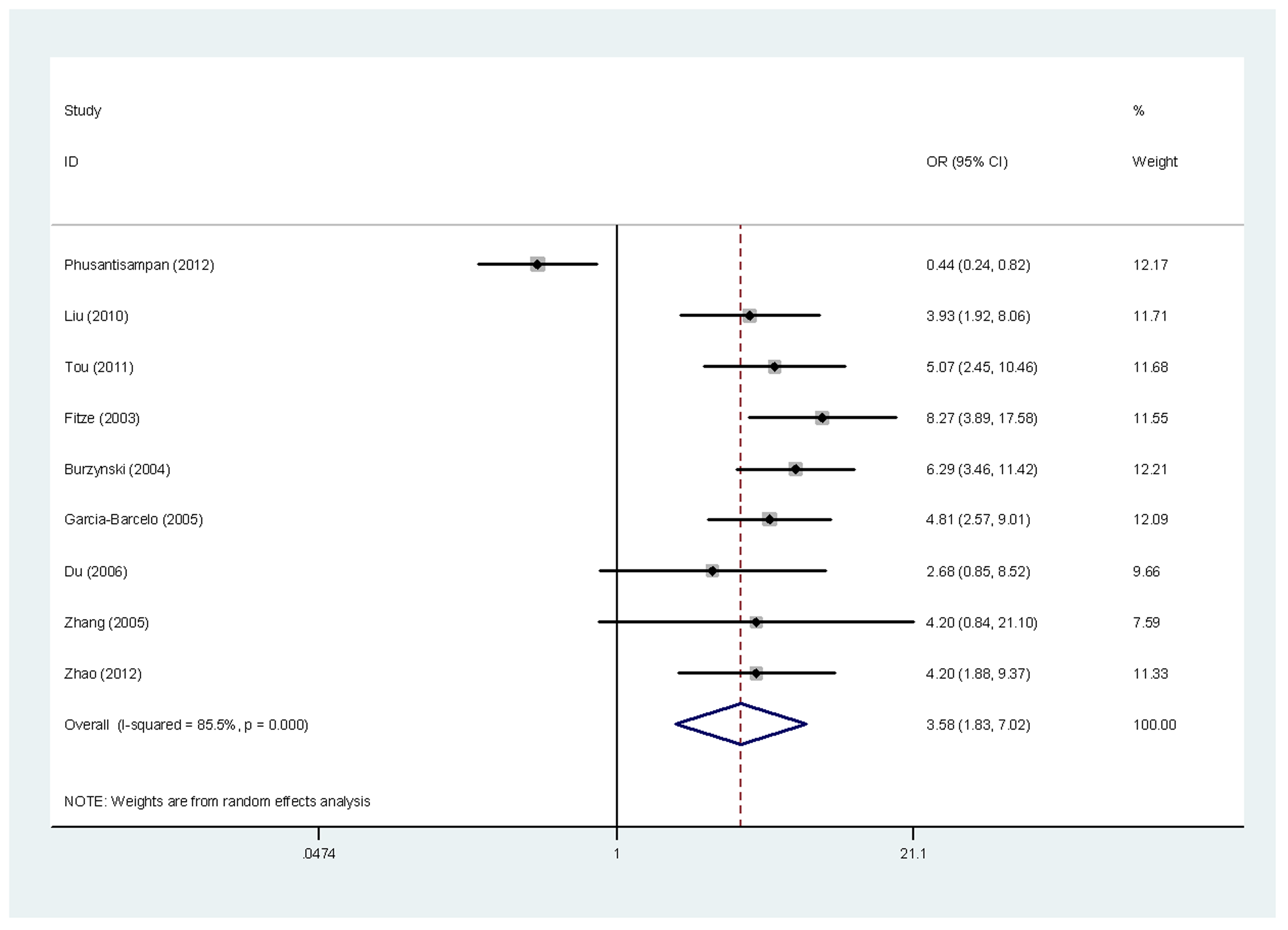


**Figure2. Meta-analysis with a radom-effect model for the ORs of HSCR risk associated with rs1800858 AA+AG vs. GG**


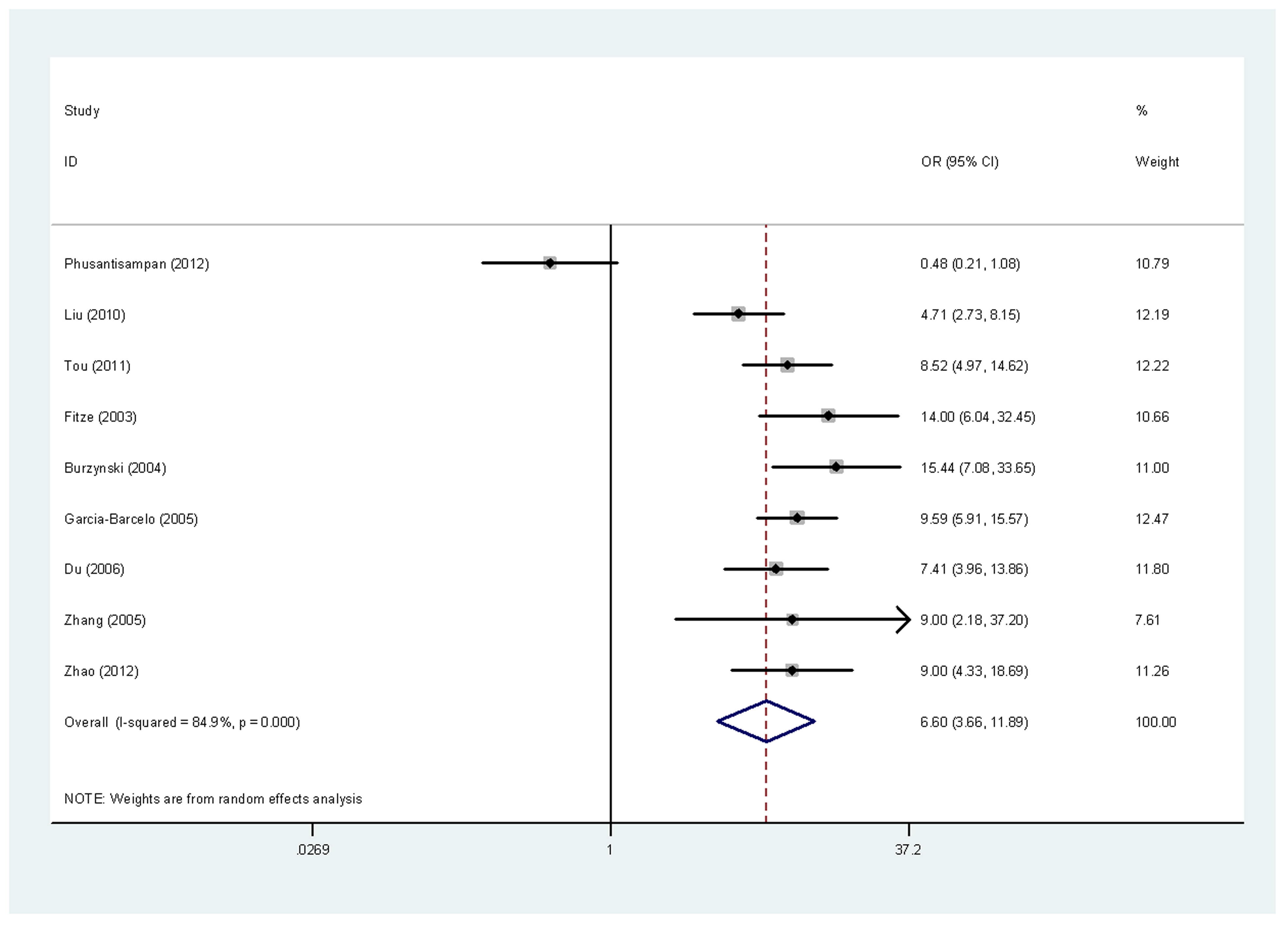


**Figure 3. Meta-analysis with a radom-effect model for the ORs of HSCR risk associated with rs1800858 AA vs. GG+AG**


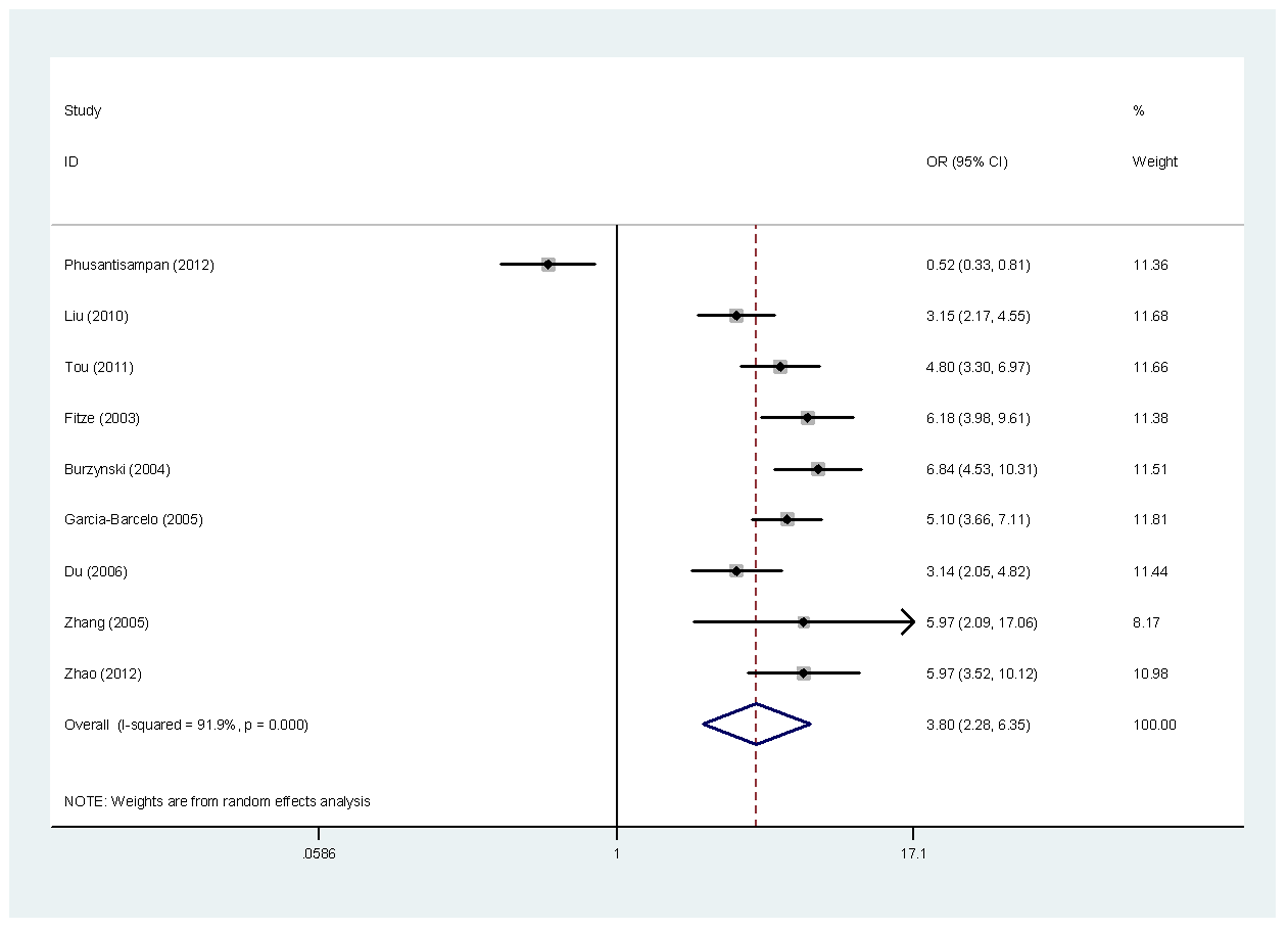


**Figure 4. Meta-analysis with a radom-effect model for the ORs of HSCR risk associated with rs1800858 A vs. G**


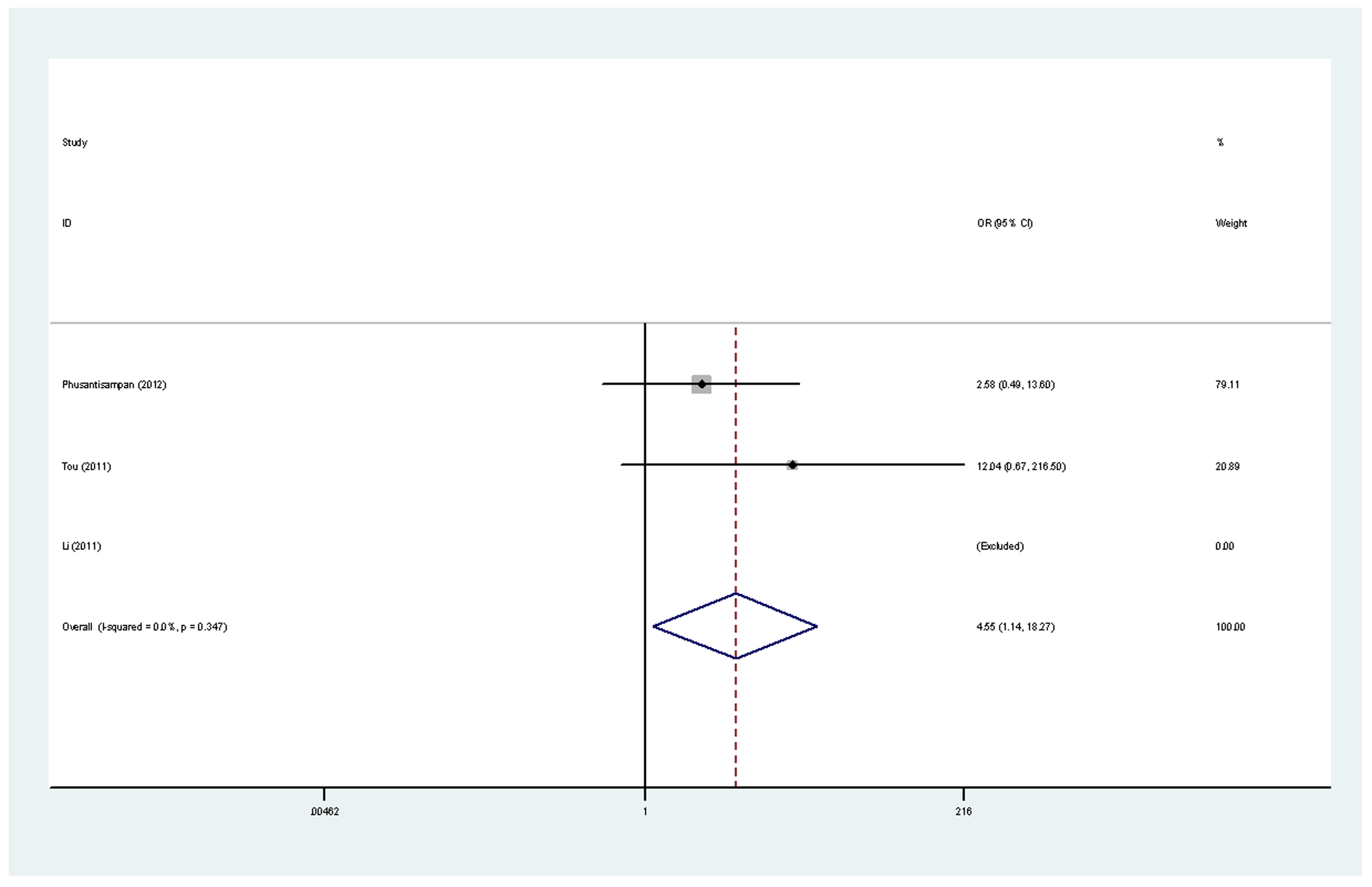


**Figure 5. Meta-analysis with a fixed-effect model for the ORs of HSCR risk associated with rs1800860 GG vs. AA**


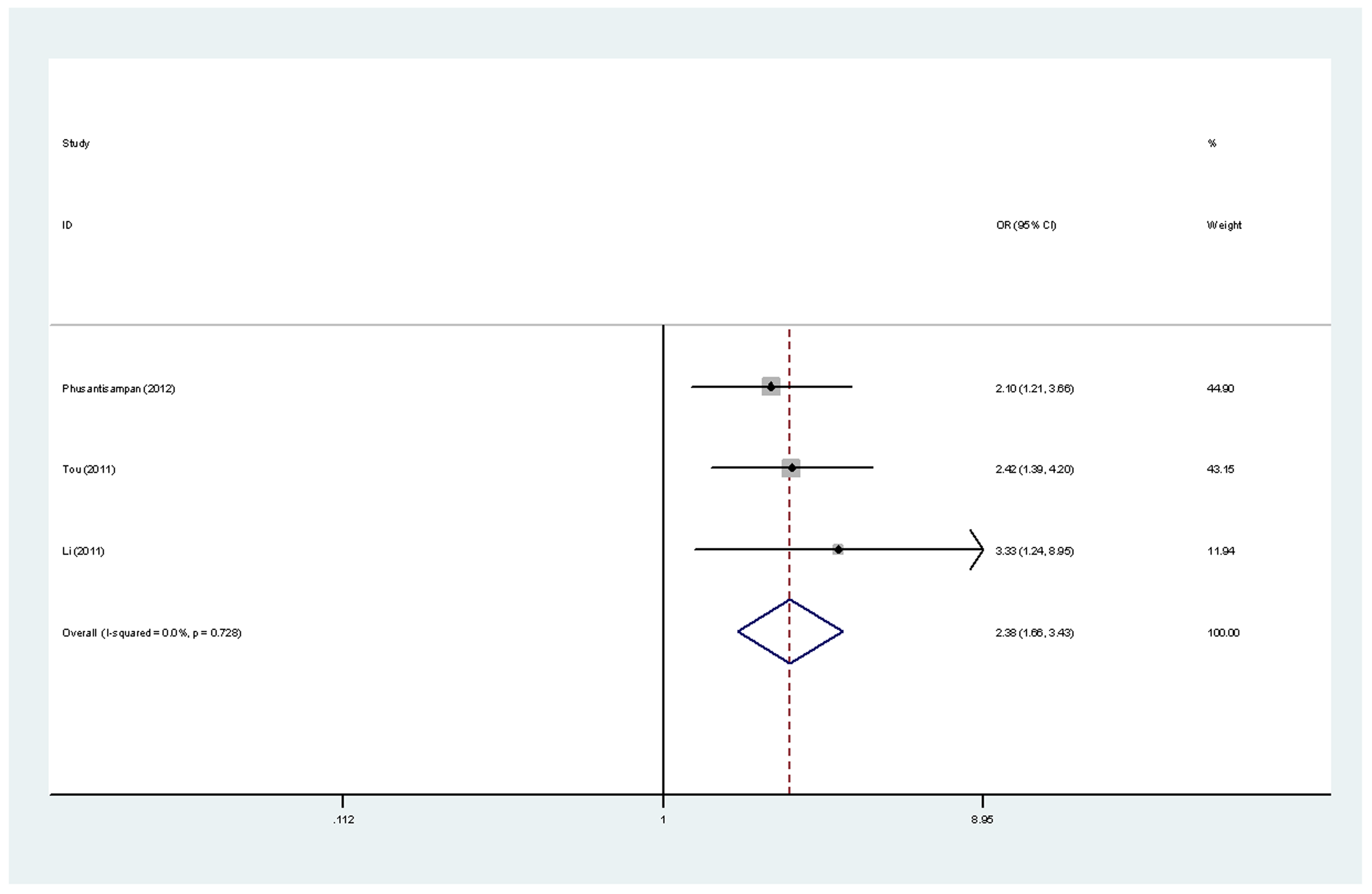


**Figure 6. Meta-analysis with a fixed-effect model for the ORs of HSCR risk associated with rs1800860 GG vs. AA+GA**


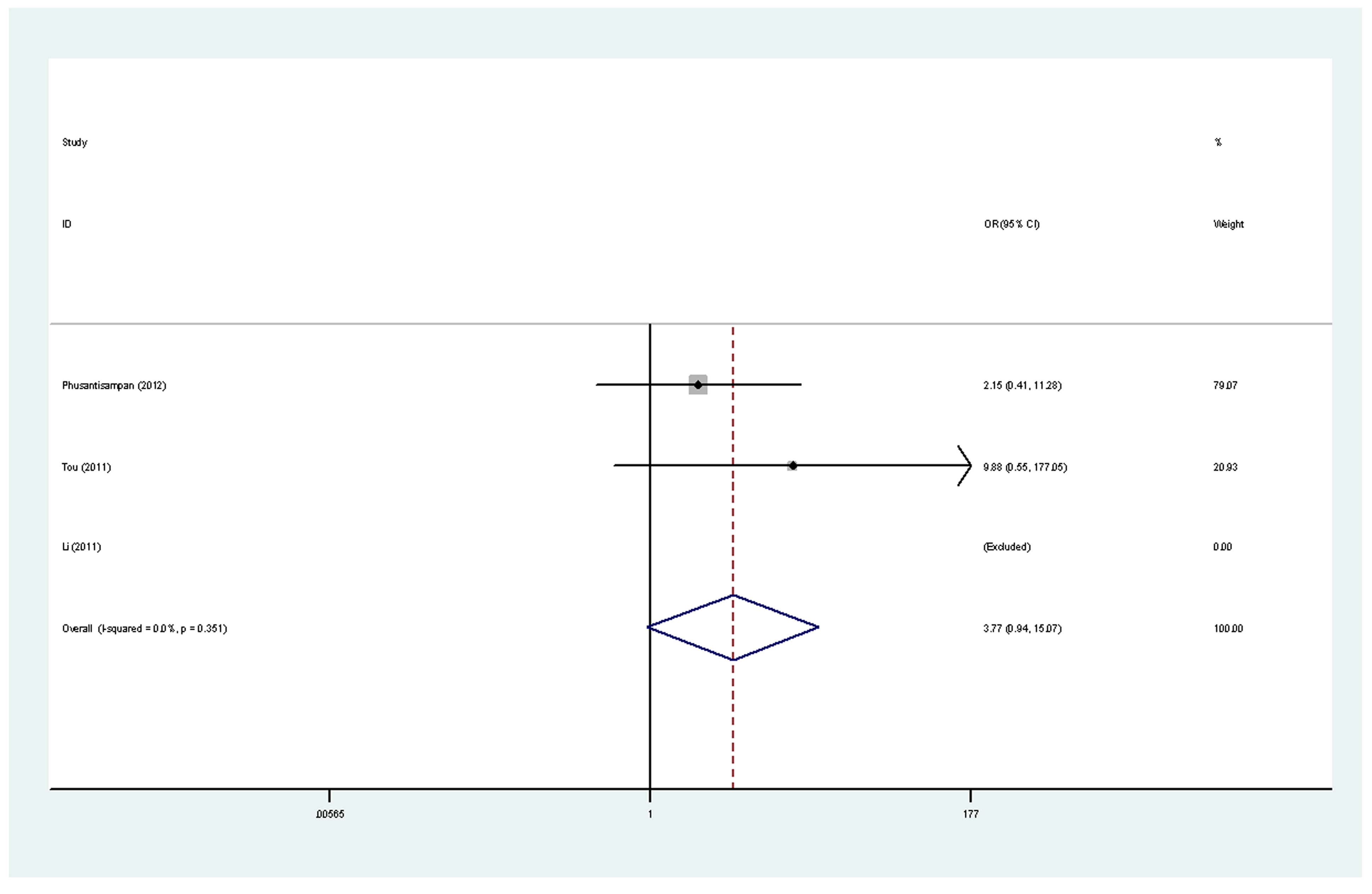


**Figure 7. Meta-analysis with a fixed-effect model for the ORs of HSCR risk associated with rs1800860 GG+GA vs. AA**


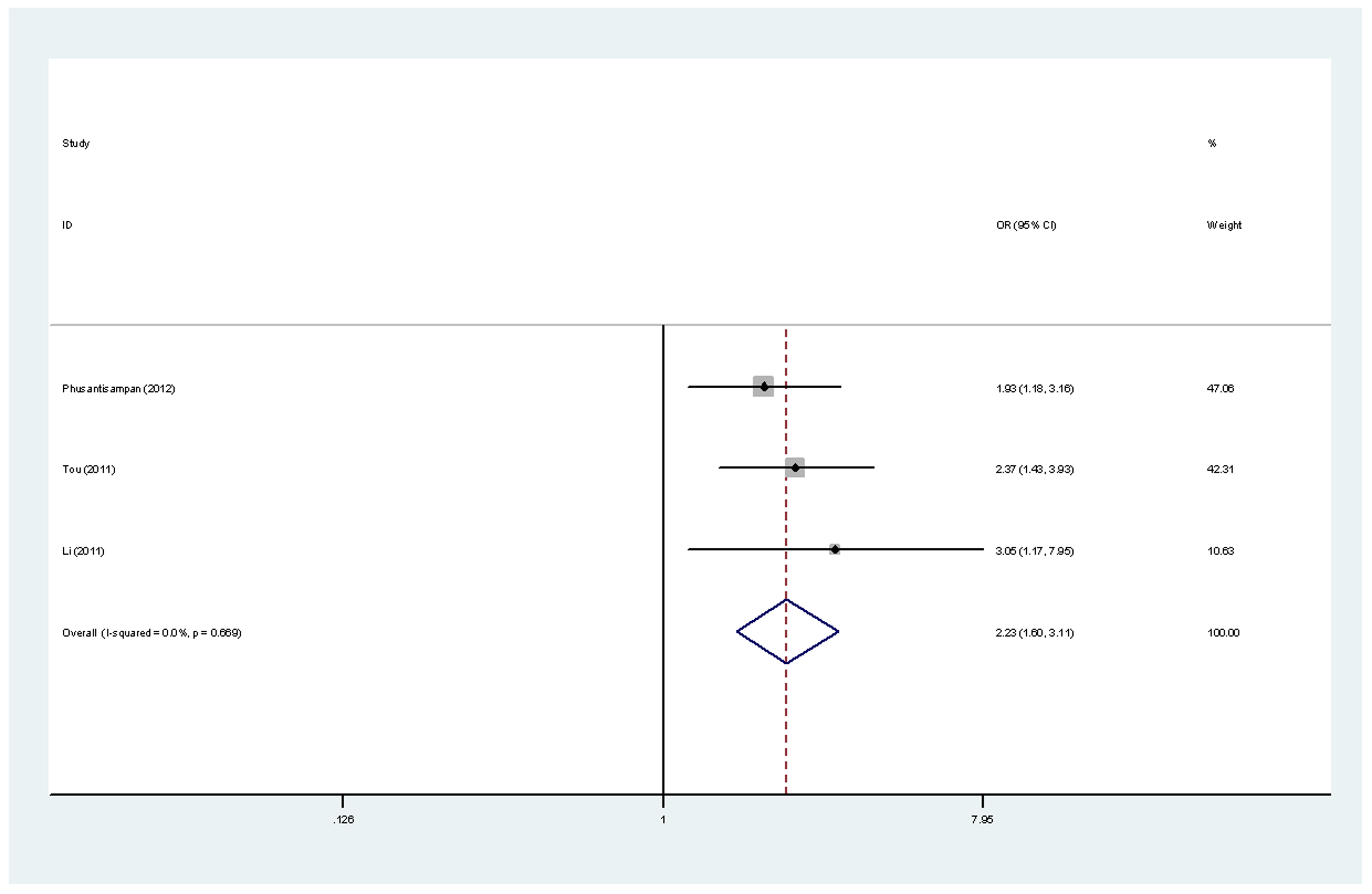


**Figure8. Meta-analysis with a fixed-effect model for the ORs of HSCR risk associated with rs1800860 G vs. A**


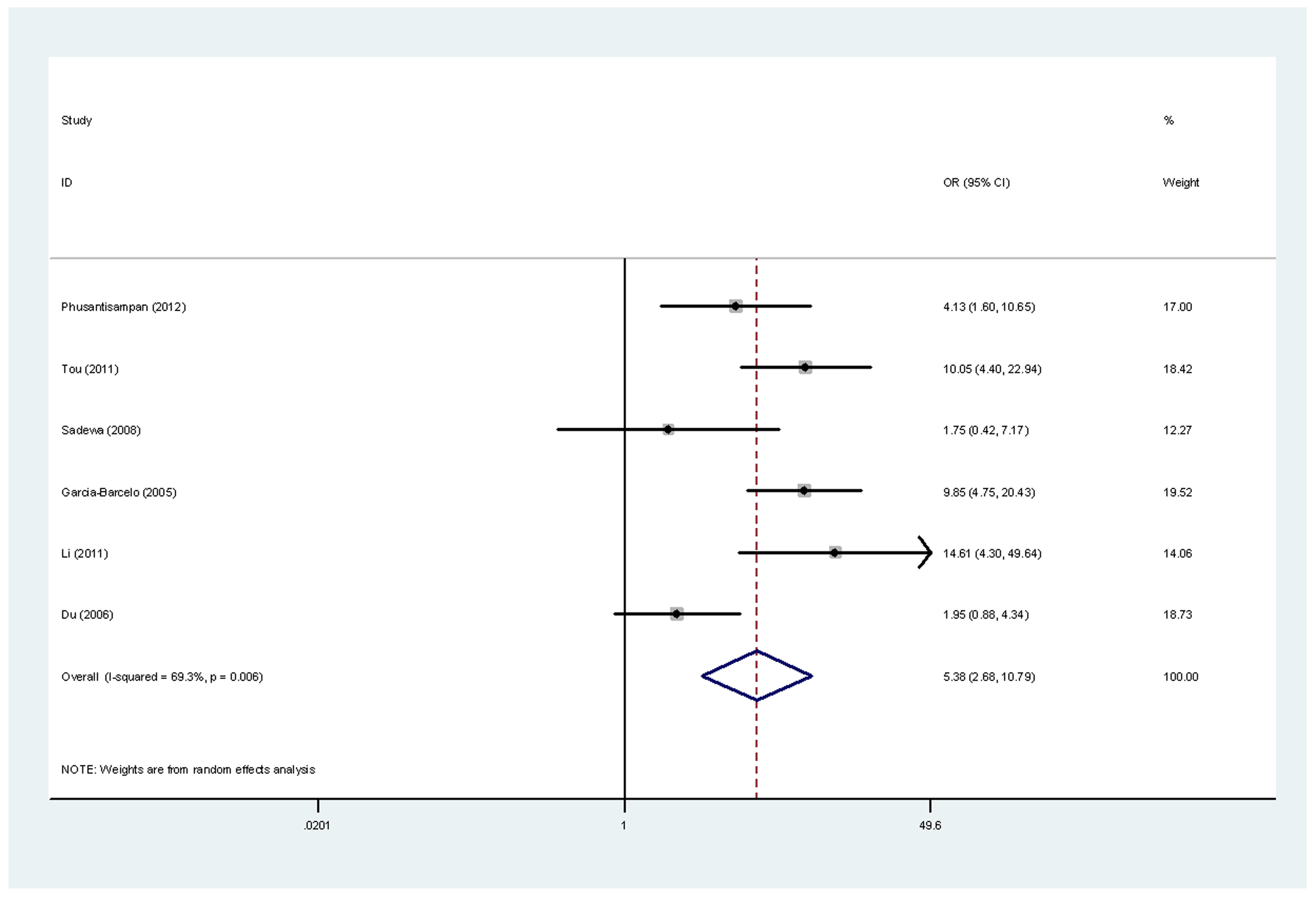


**Figure 9. Meta-analysis with a radom-effect model for the ORs of HSCR risk associated with rs1800861 GG vs. TT**


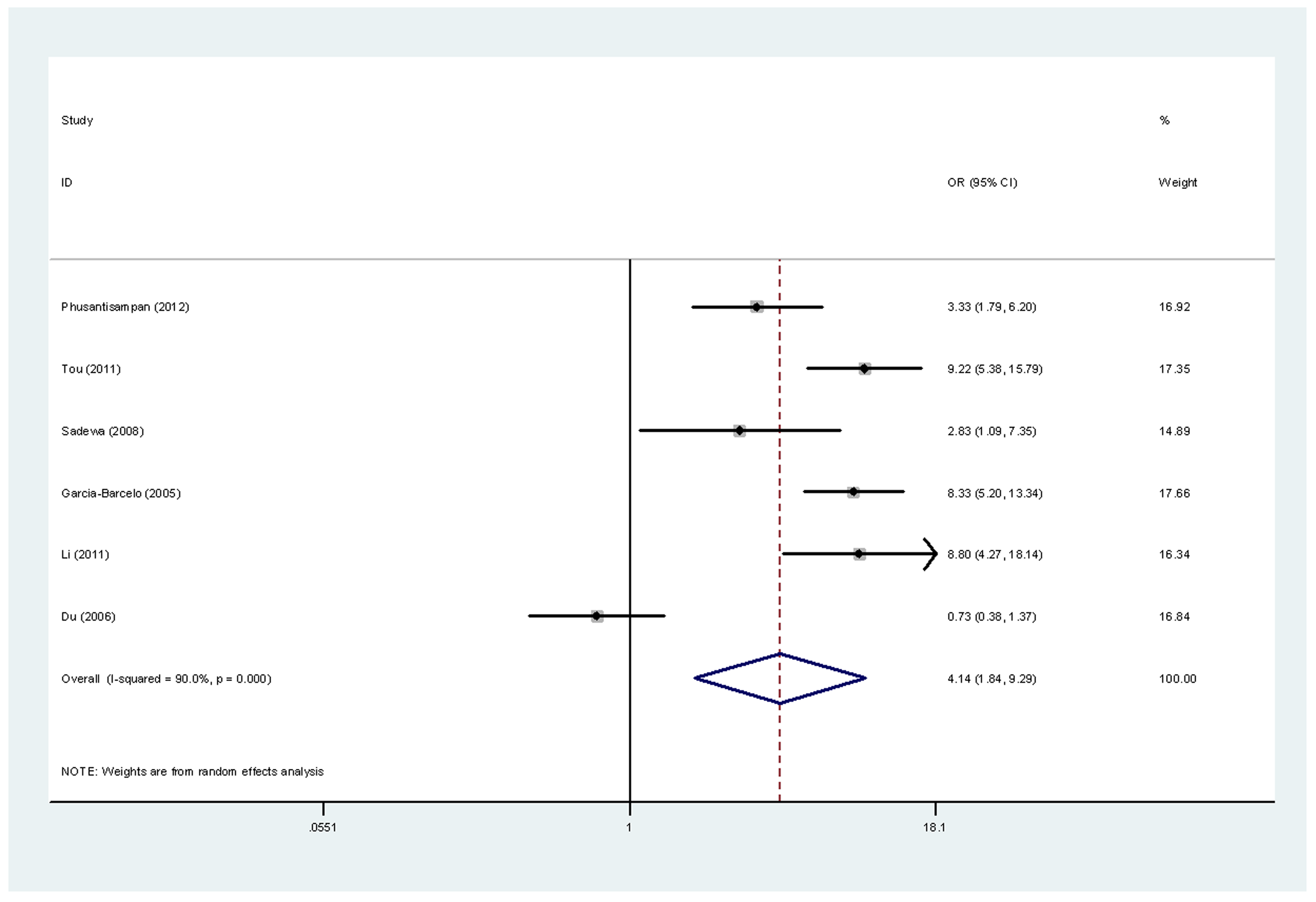


**Figure 10. Meta-analysis with a radom-effect model for the ORs of HSCR risk associated with rs1800861 GG vs. TT+TG**


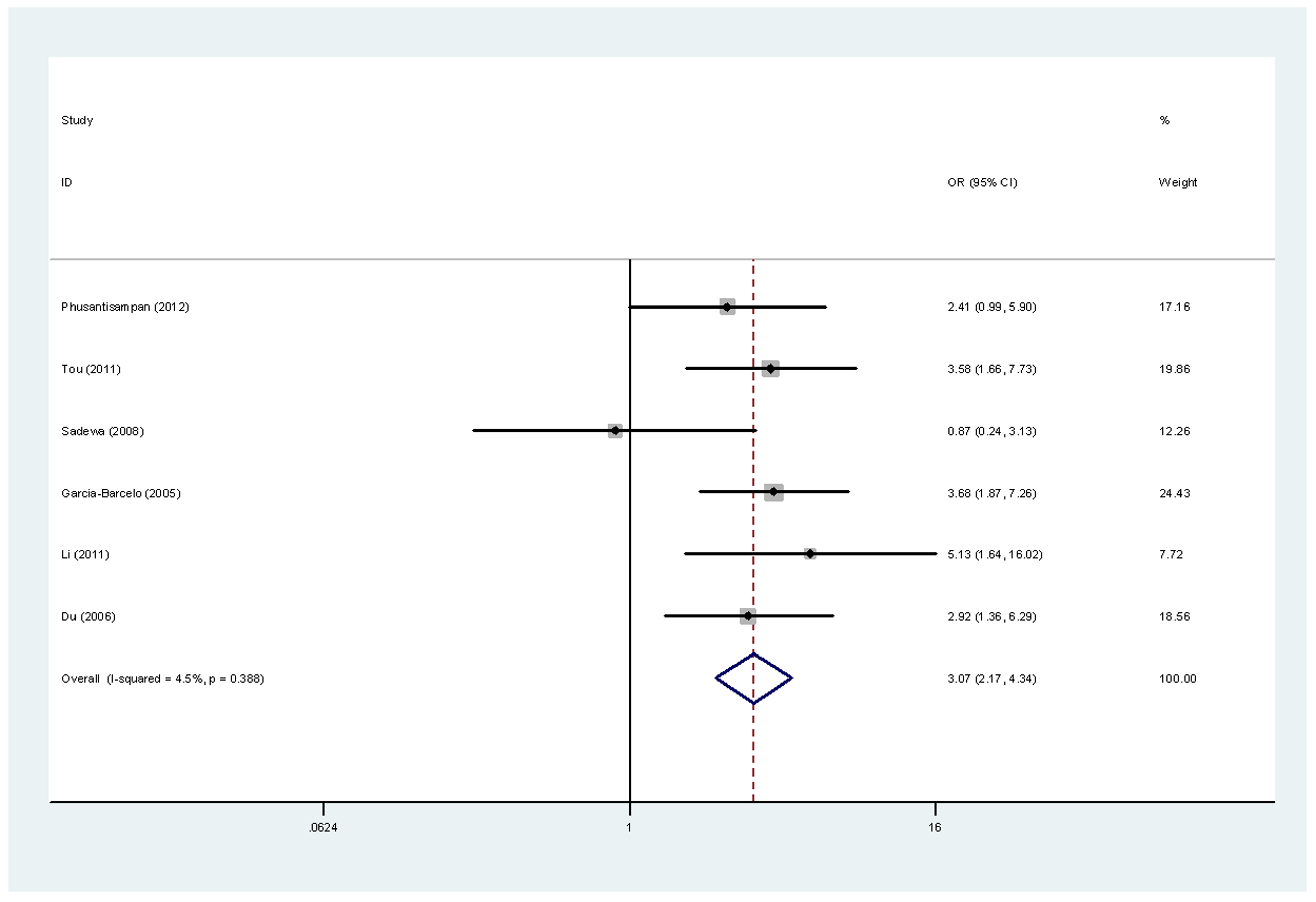


**Figure 11. Meta-analysis with a fixed-effect model for the ORs of HSCR risk associated with rs1800861 GG+TG vs. TT**


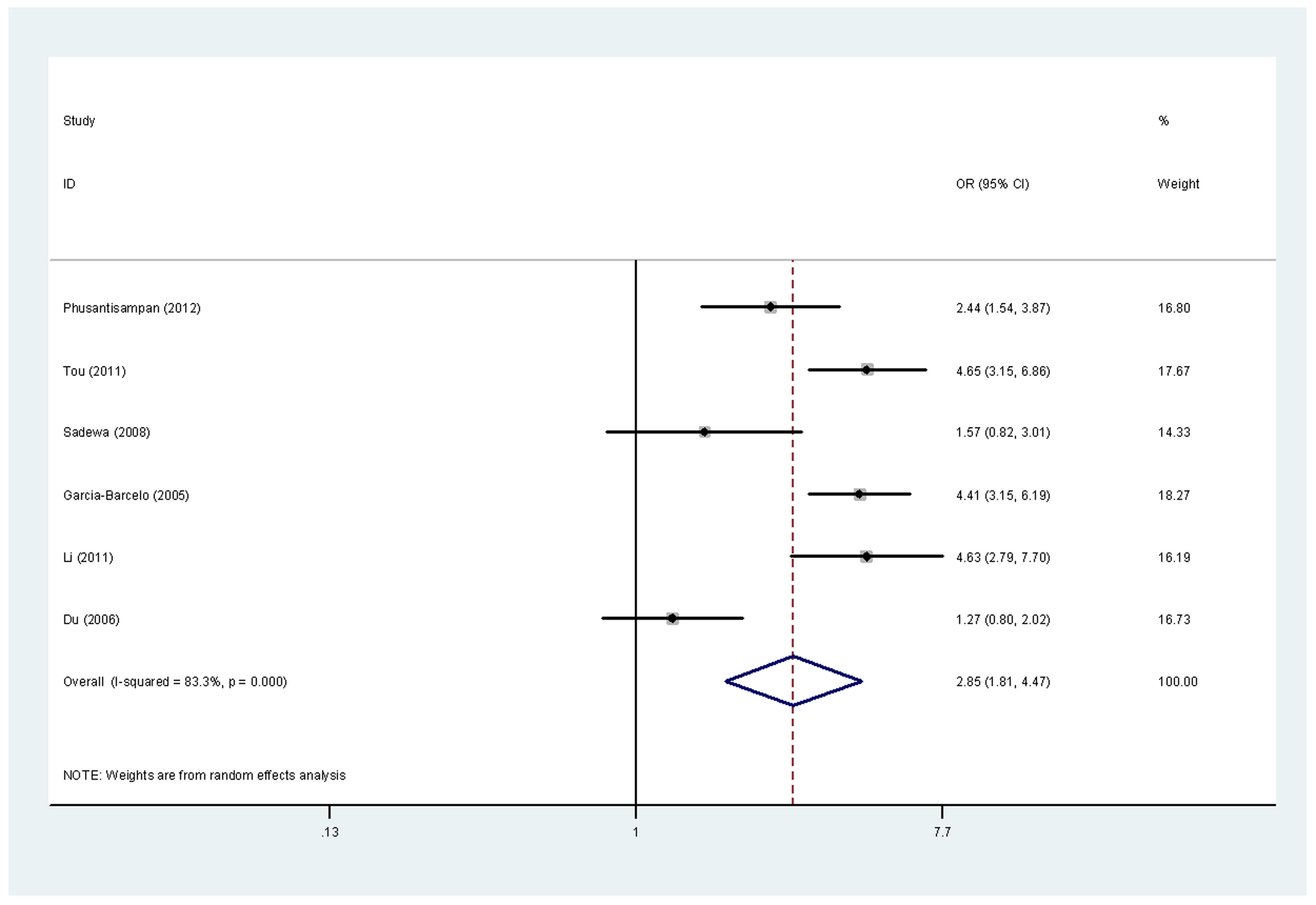


**Figure 12. Meta-analysis with a radom-effect model for the ORs of HSCR risk associated with rs1800861 G vs. T**


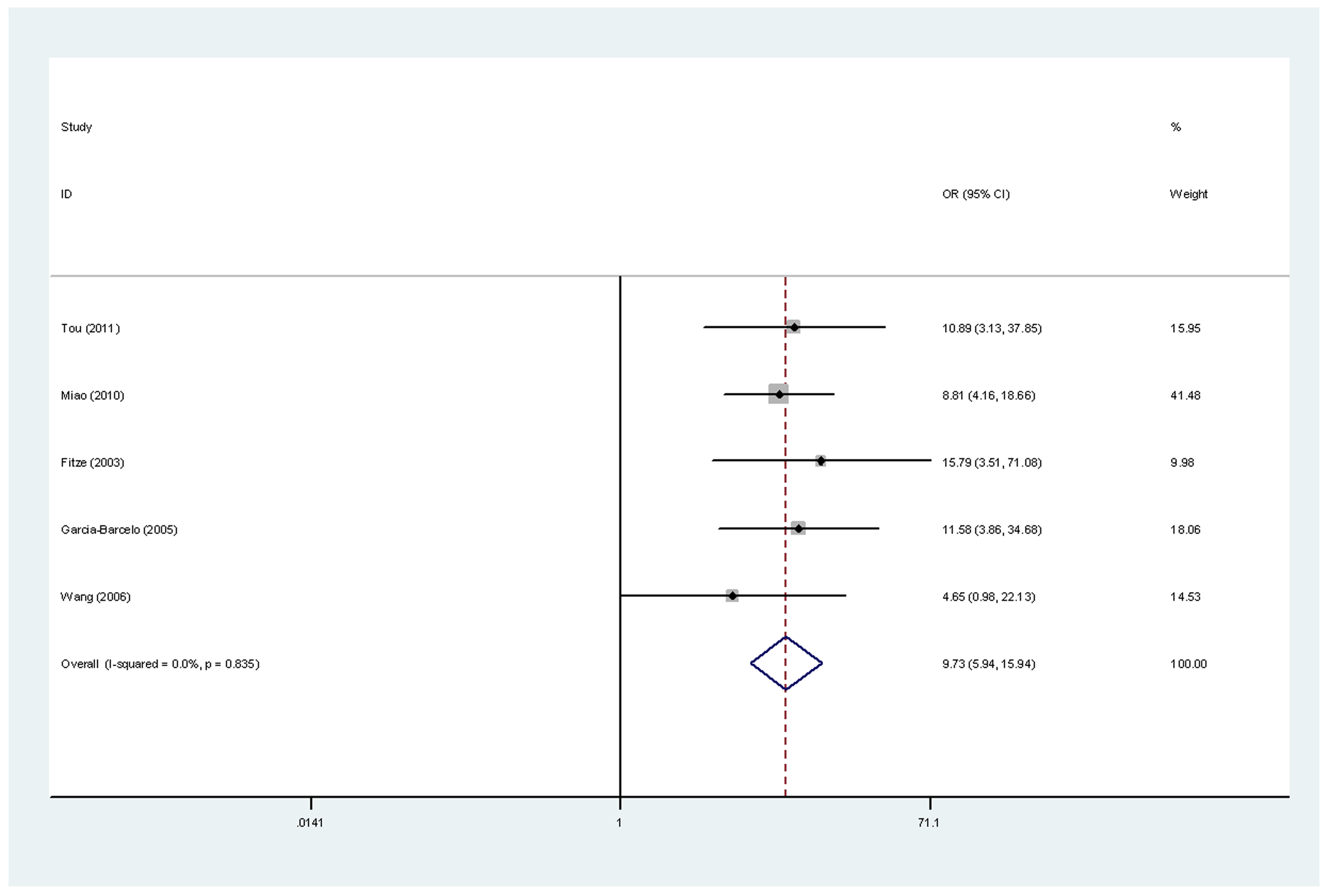


**Figure13. Meta-analysis with a fixed-effect model for the ORs of HSCR risk associated with rs10900297 CC vs. AA**


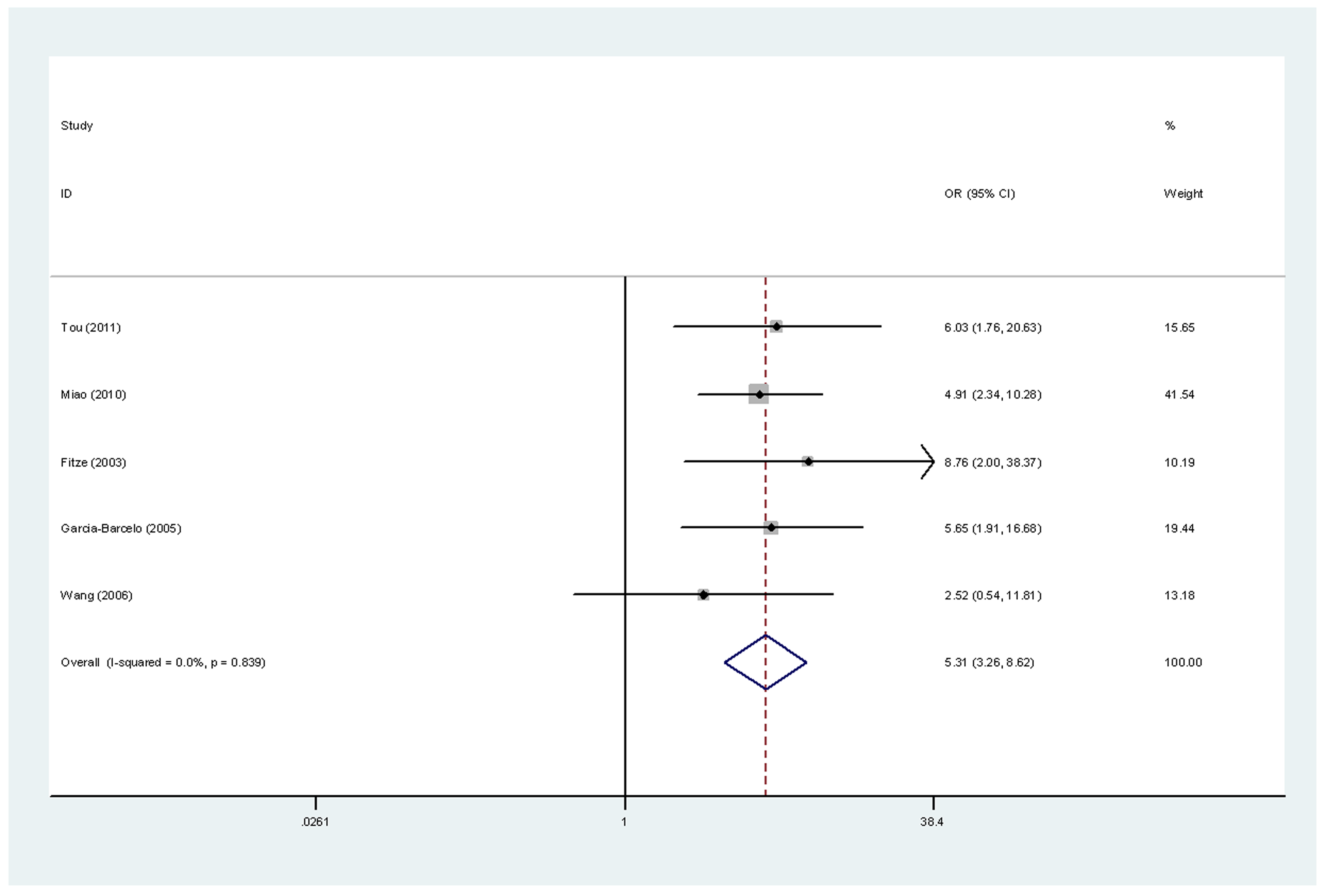


**Figure 14. Meta-analysis with a fixed-effect model for the ORs of HSCR risk associated with rs10900297 CC+AC vs. AA**


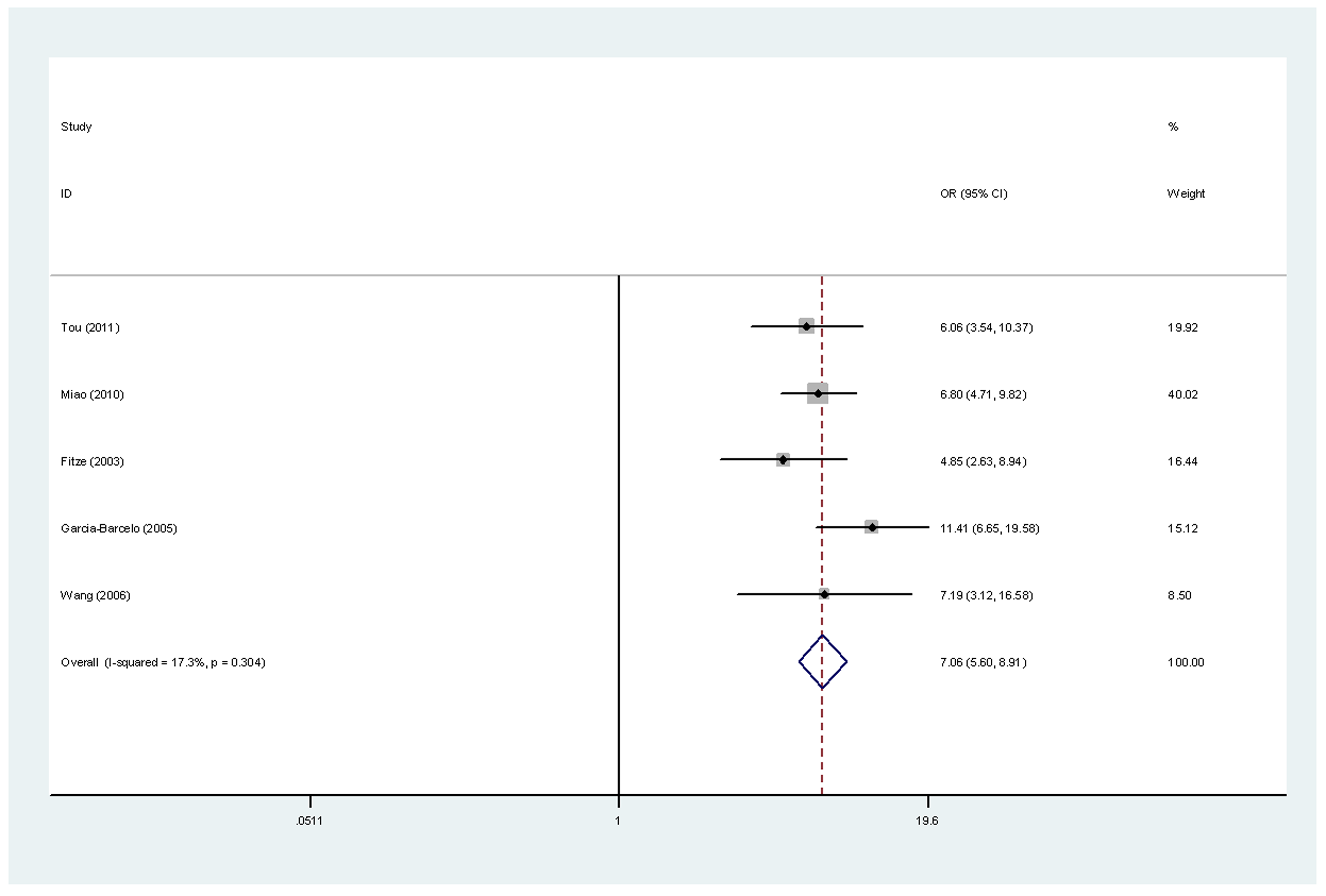


**Figure15. Meta-analysis with a fixed-effect model for the ORs of HSCR risk associated with rs10900297 CC vs. AA+AC**


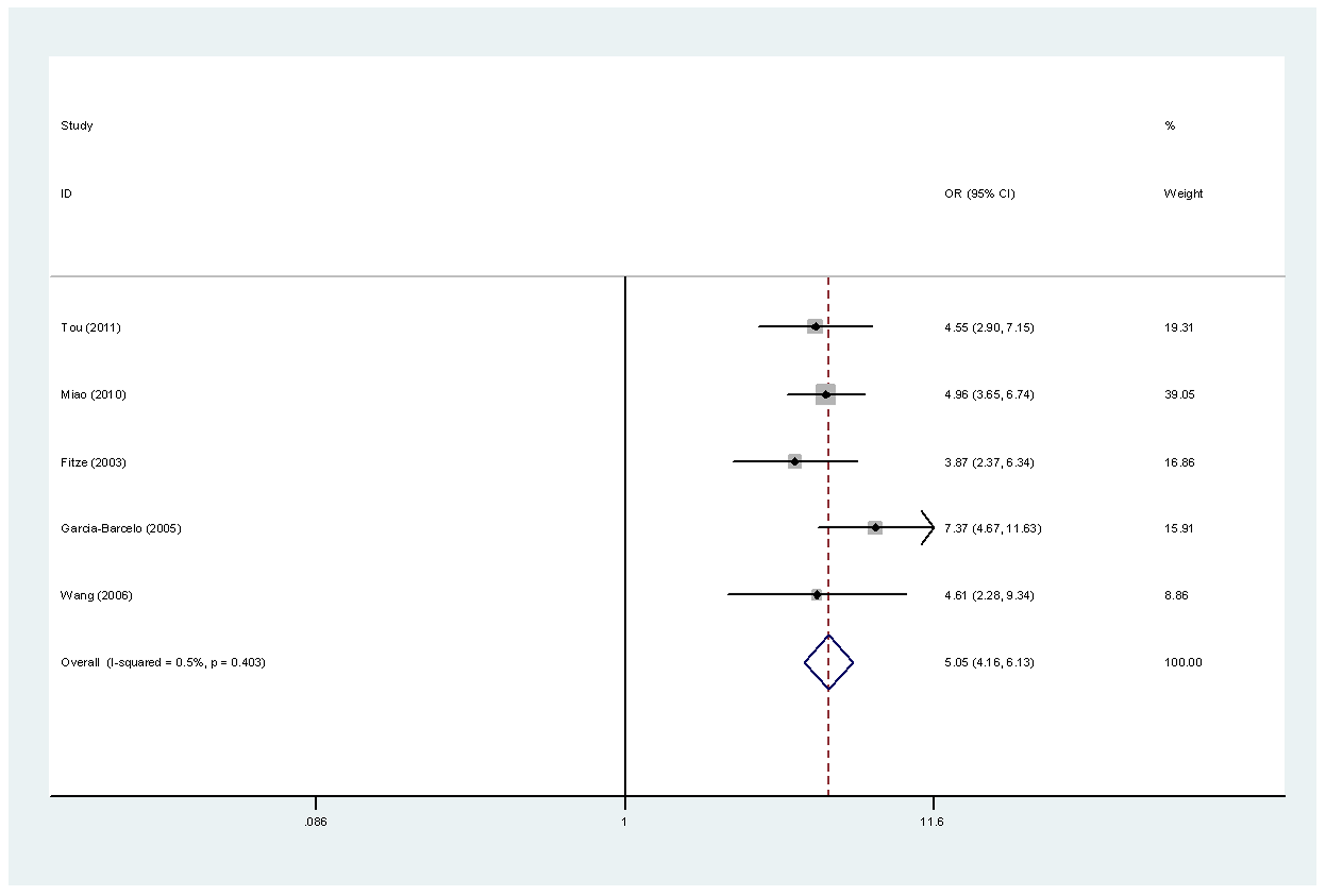


**Figure 16. Meta-analysis with a fixed-effect model for the ORs of HSCR risk associated with rs10900297 C vs. A**


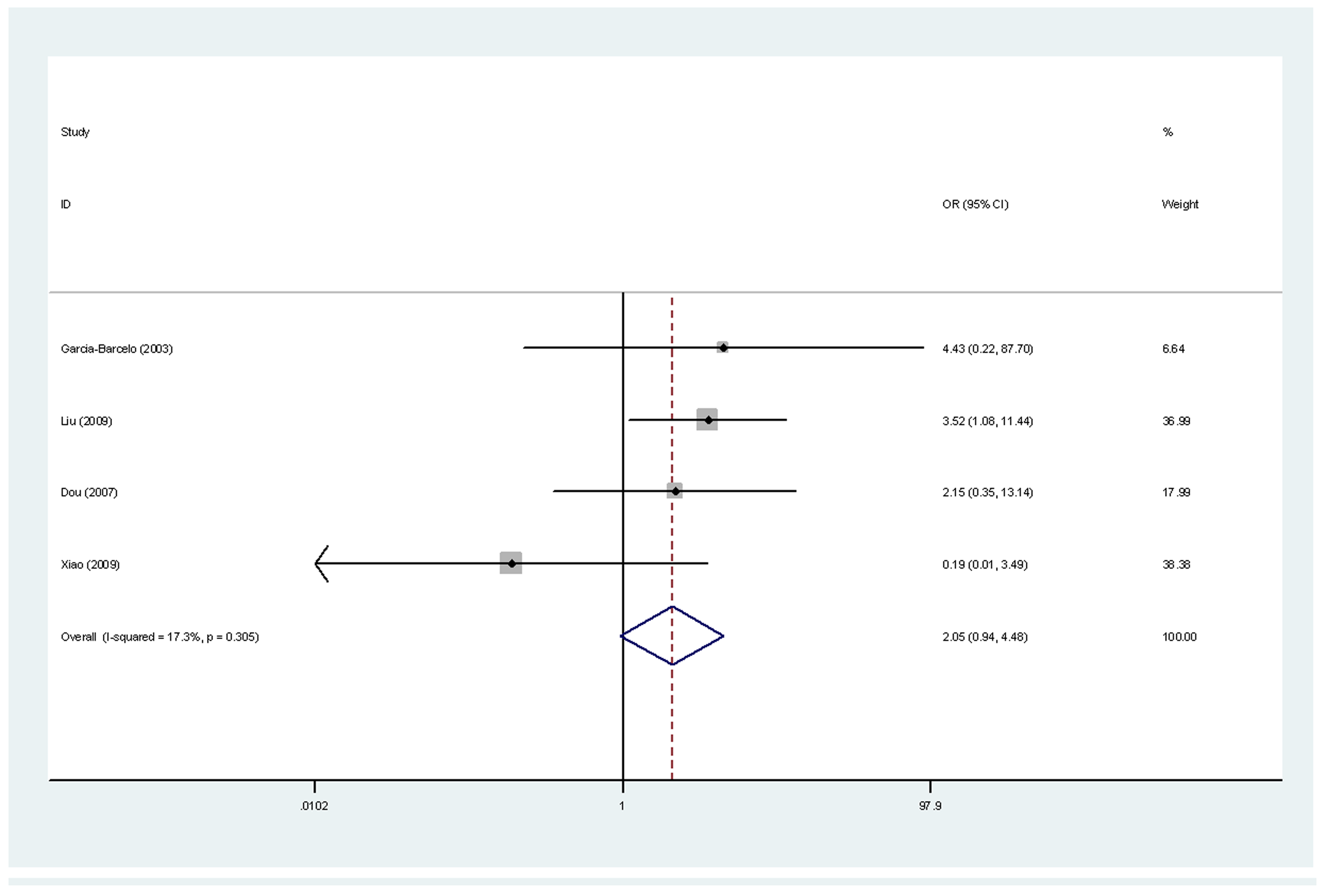


**Figure 17. Meta-analysis with a fixed-effect model for the ORs of HSCR risk associated with rs26847582 GG vs AA**


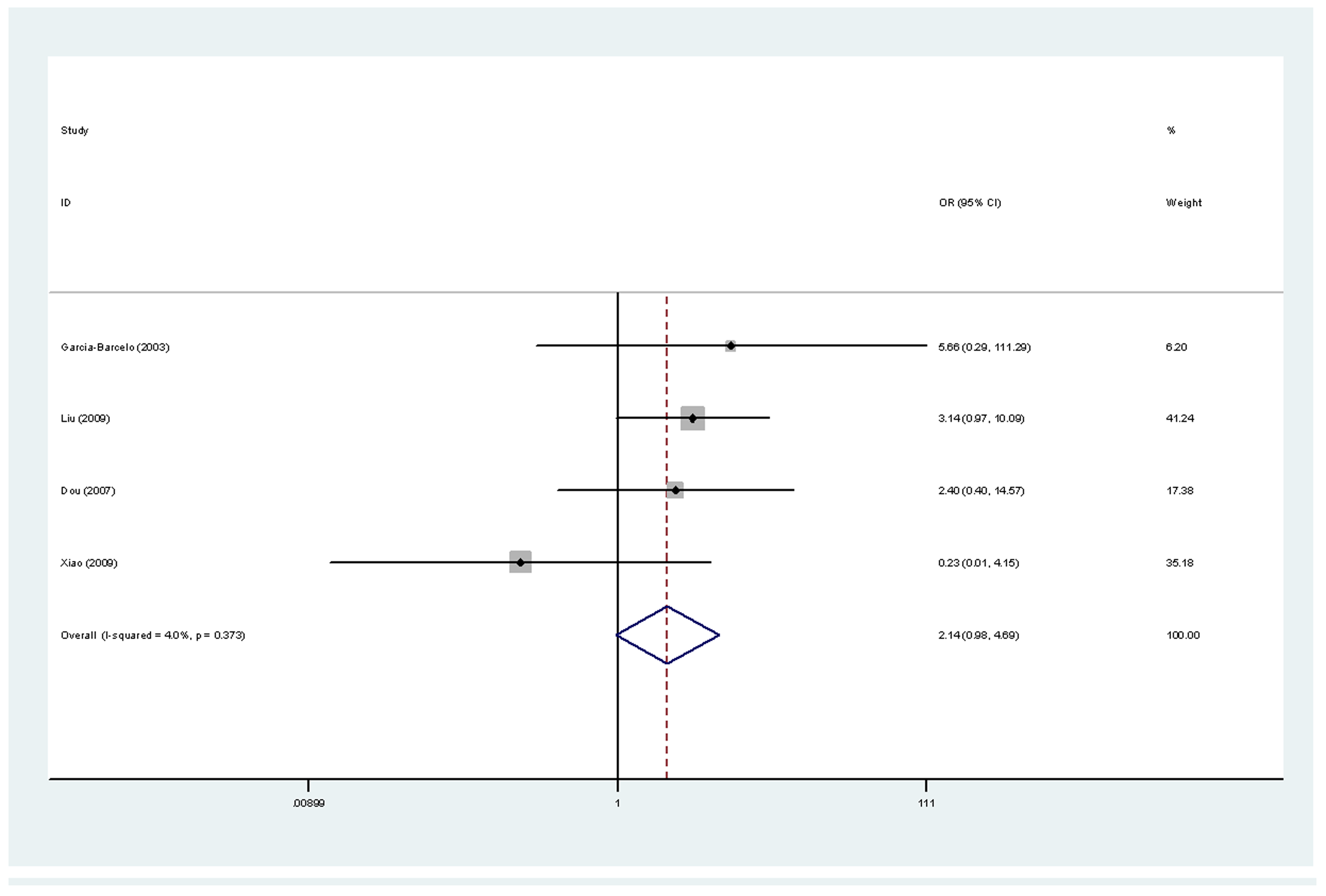


**Figure 18. Meta-analysis with a fixed-effect model for the ORs of HSCR risk associated with rs26847582 GG vs GA+AA**


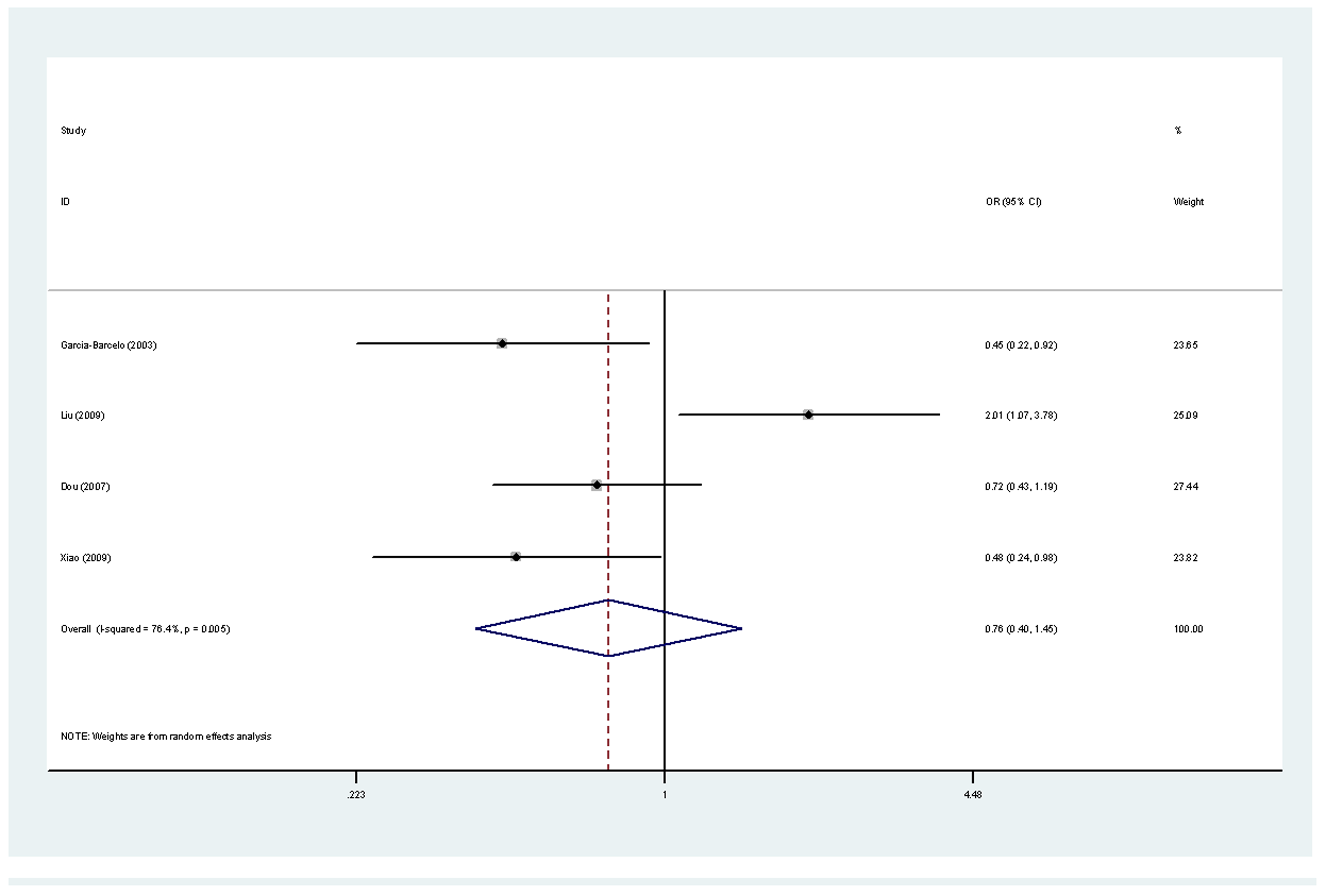


**Figure 19. Meta-analysis with a radom-effect model for the ORs of HSCR risk associated with rs26847582 GG+GA vs AA**


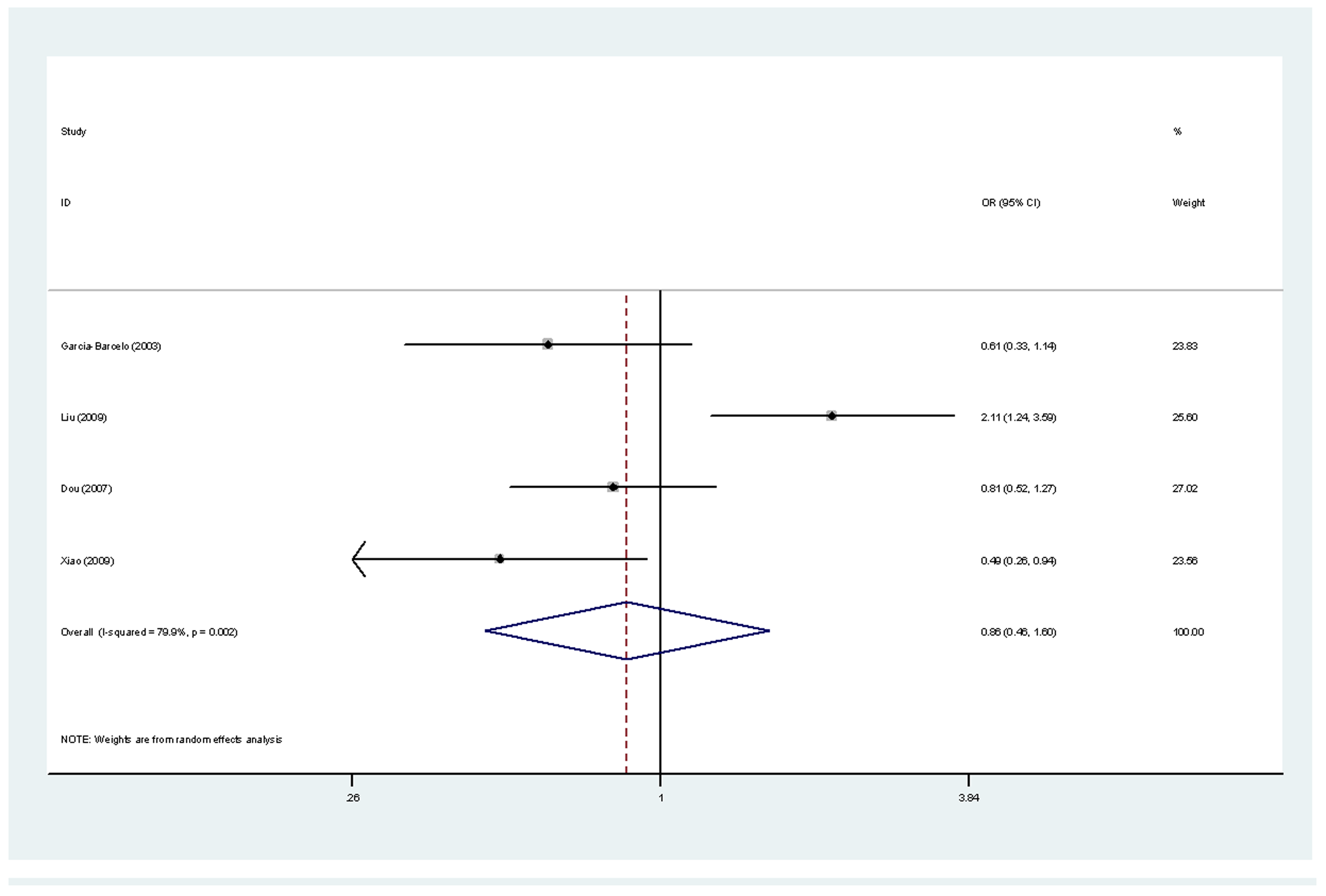


**Figure 20. Meta-analysis with a radom-effect model for the ORs of HSCR risk associated with rs26847582 G vs A**


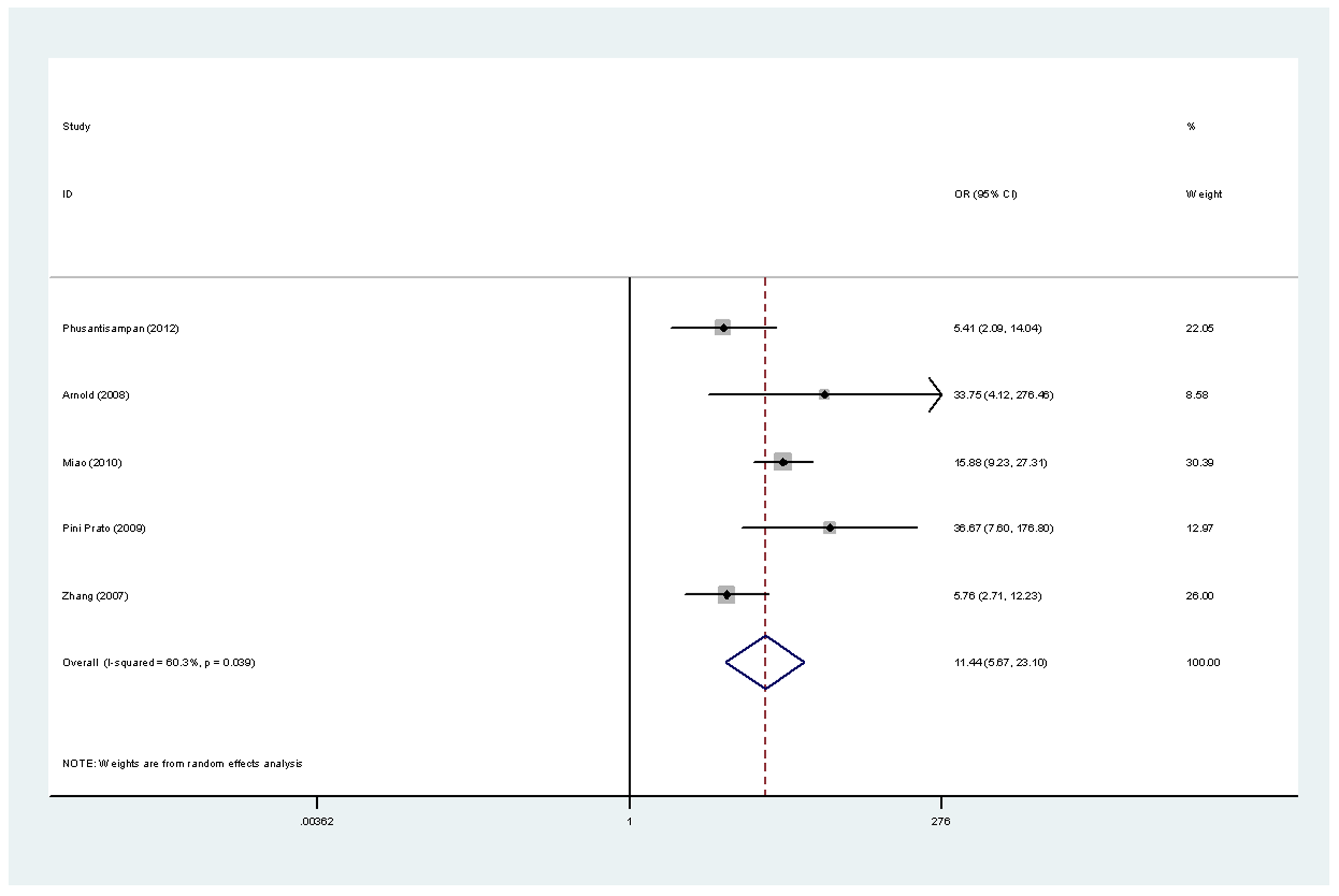


**Figure 21. Meta-analysis with a radom-effect model for the ORs of HSCR risk associated with rs2435357 TT vs. CC**


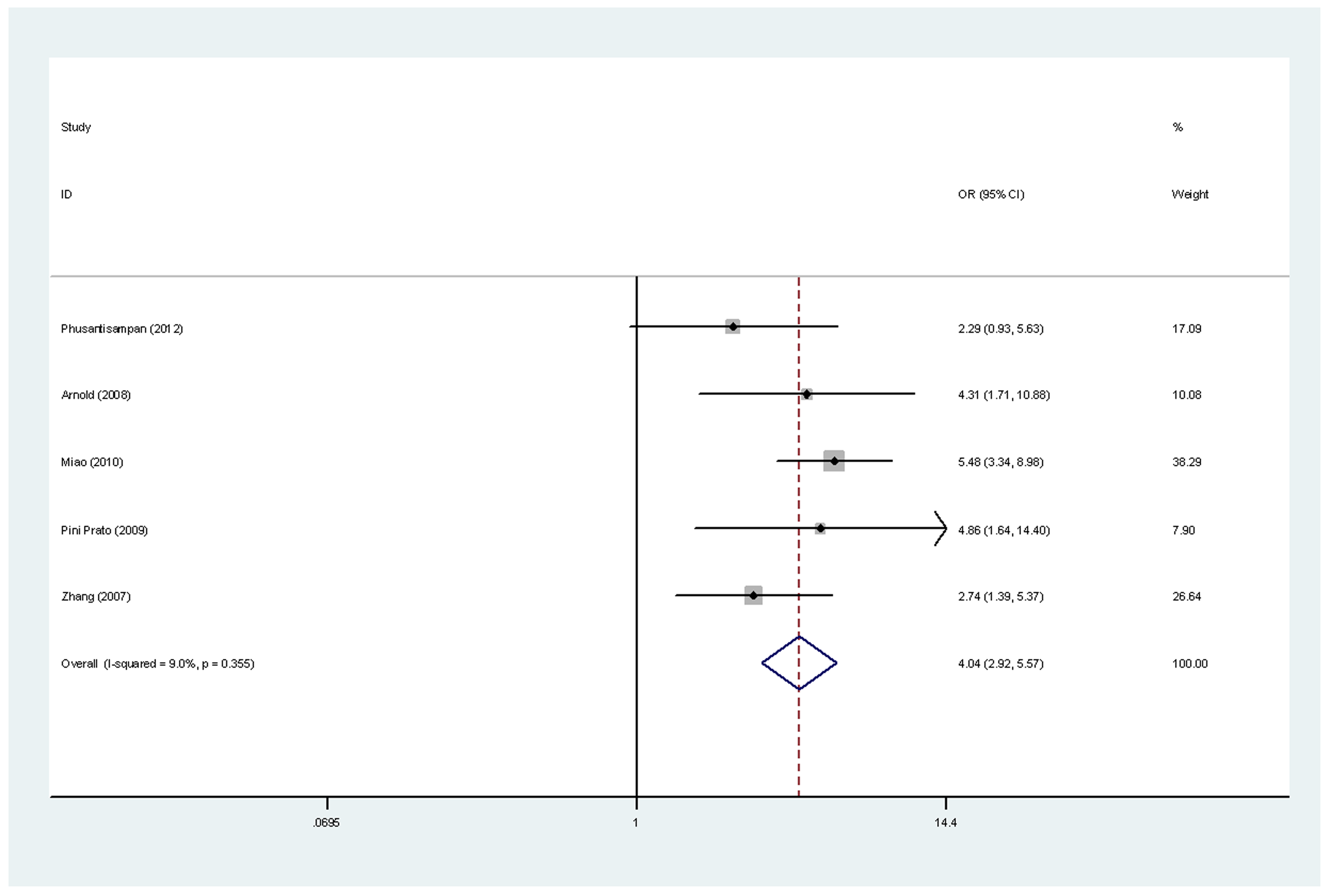


**Figure 22. Meta-analysis with a fixed-effect model for the ORs of HSCR risk associated with rs2435357 TT+TC vs. CC**


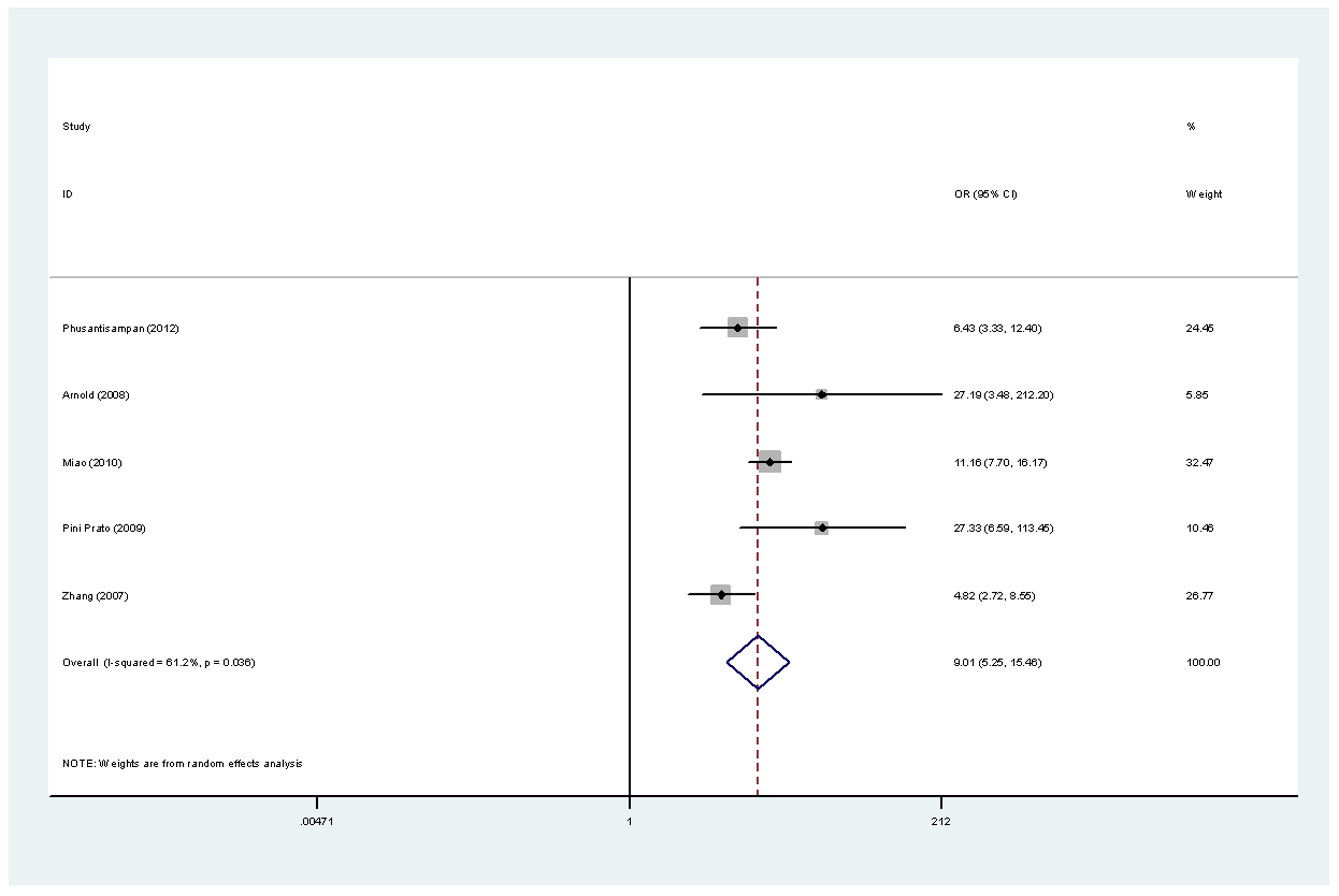


**Figure 23. Meta-analysis with a radom-effect model for the ORs of HSCR risk associated with rs2435357 TT vs. CC+TC**


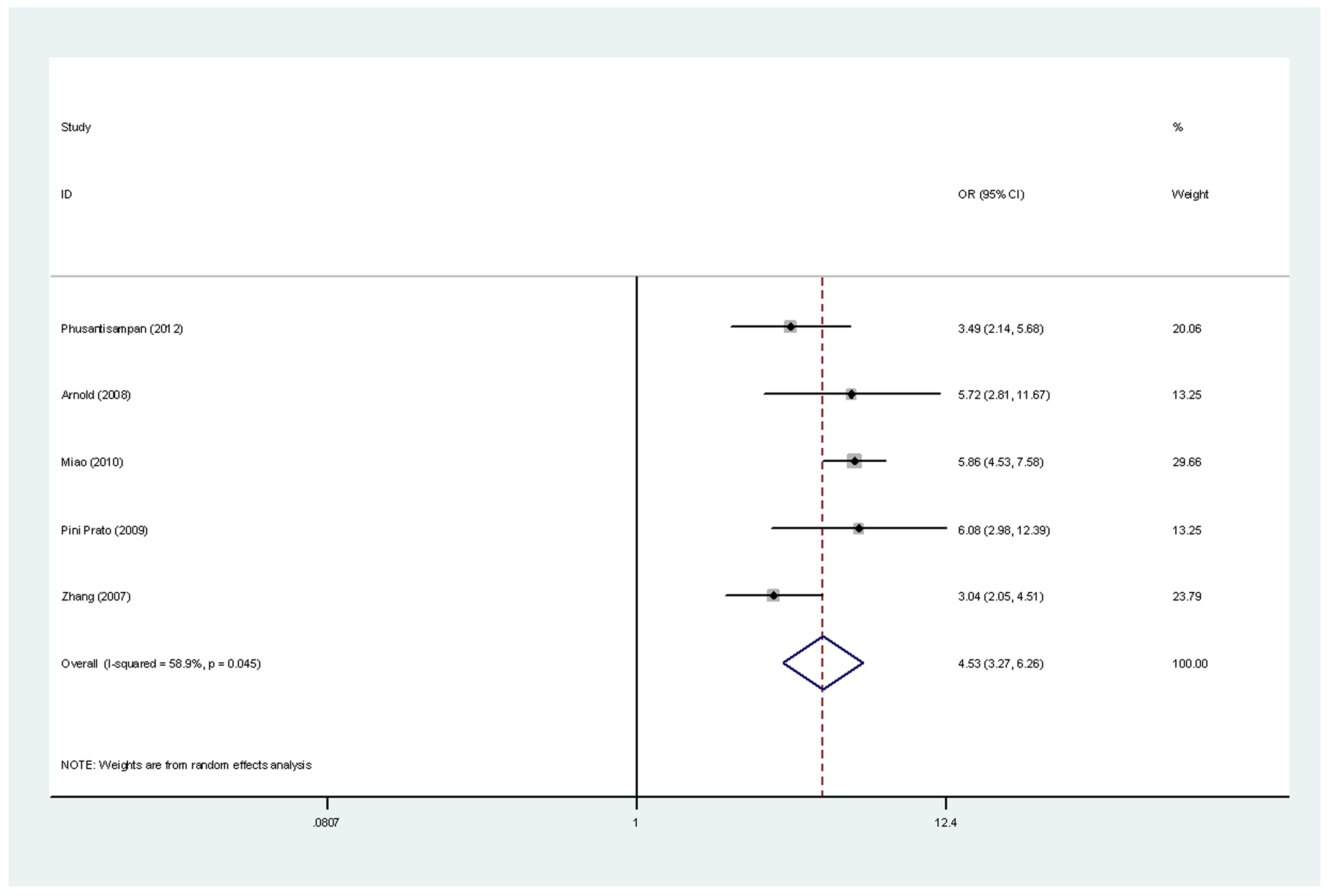


**Figure 24. Meta-analysis with a radom-effect model for the ORs of HSCR risk associated with rs2435357 T vs. C**
