# Supplementary material for: RET and PHOX2B Genetic Polymorphisms and Hirschsprung's Disease Susceptibility: A Meta-Analysis
Source: PLoS One. 2014 Mar 20;9(3):e90091. doi: 10.1371/journal.pone.0090091 (PMC3961244; doi:10.1371/journal.pone.0090091)
Supplement: Supplement S3 — Funnel plots about RET,PHOX2B gene. (DOC) [file pone.0090091.s003.doc]

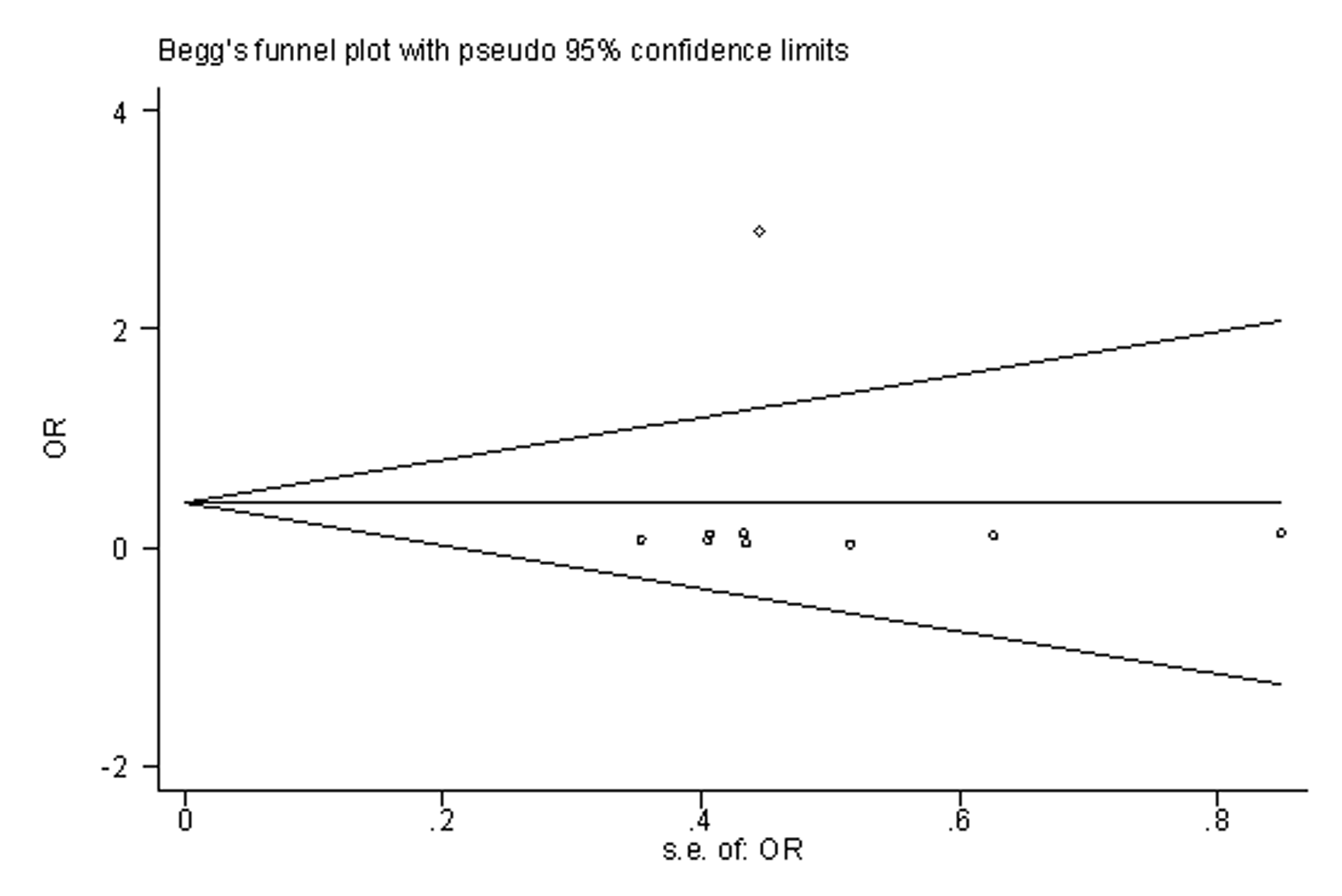


A


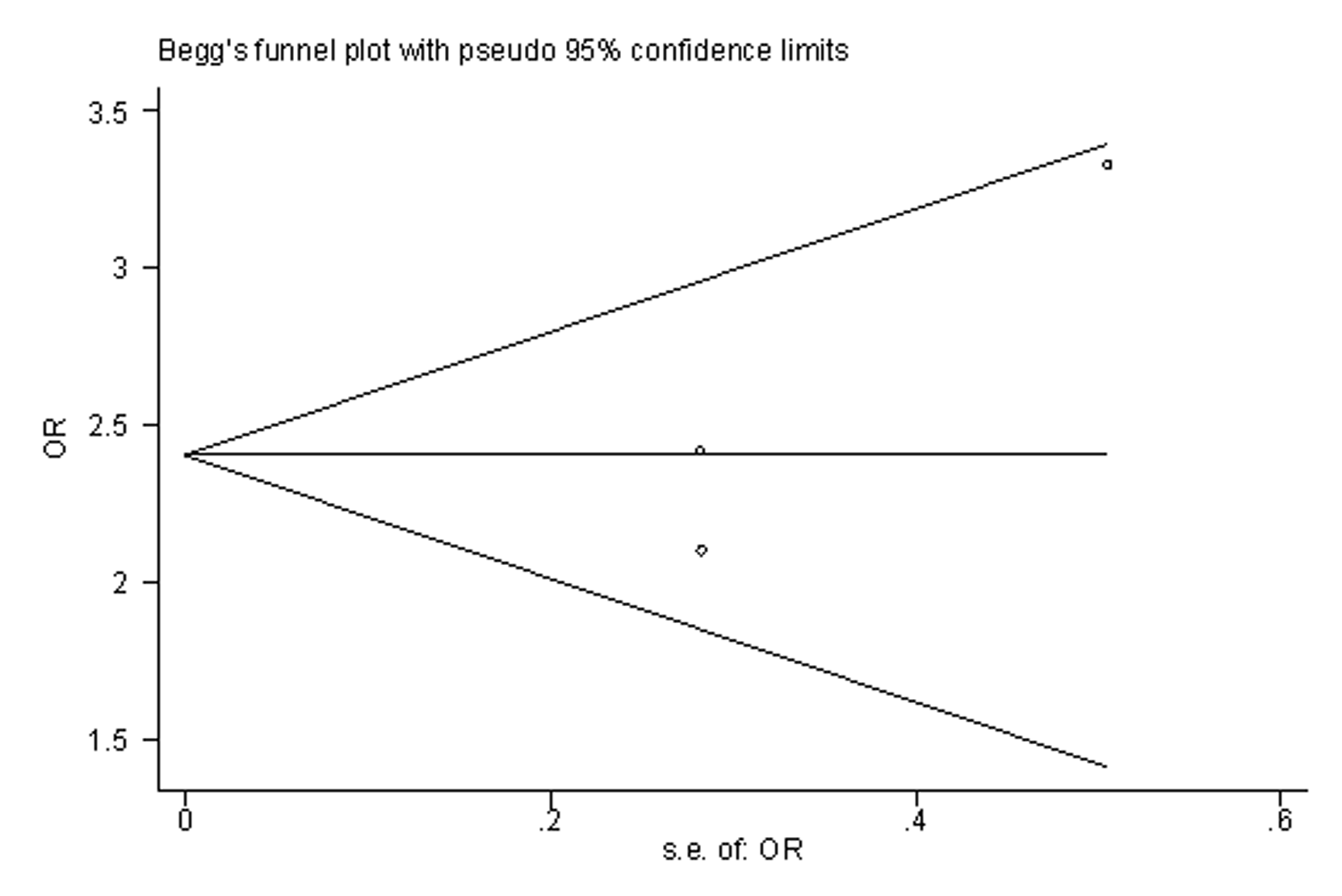


B


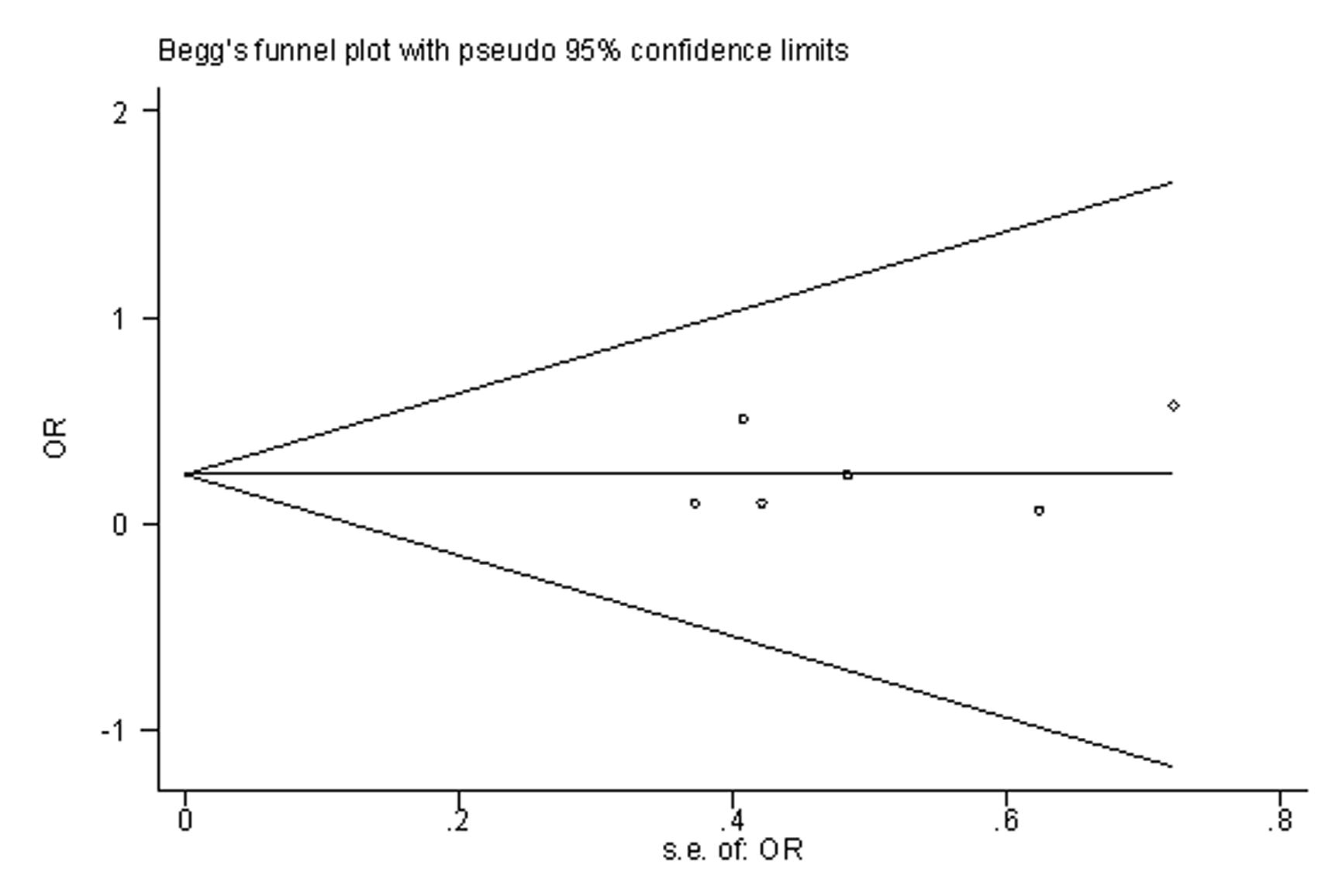


C


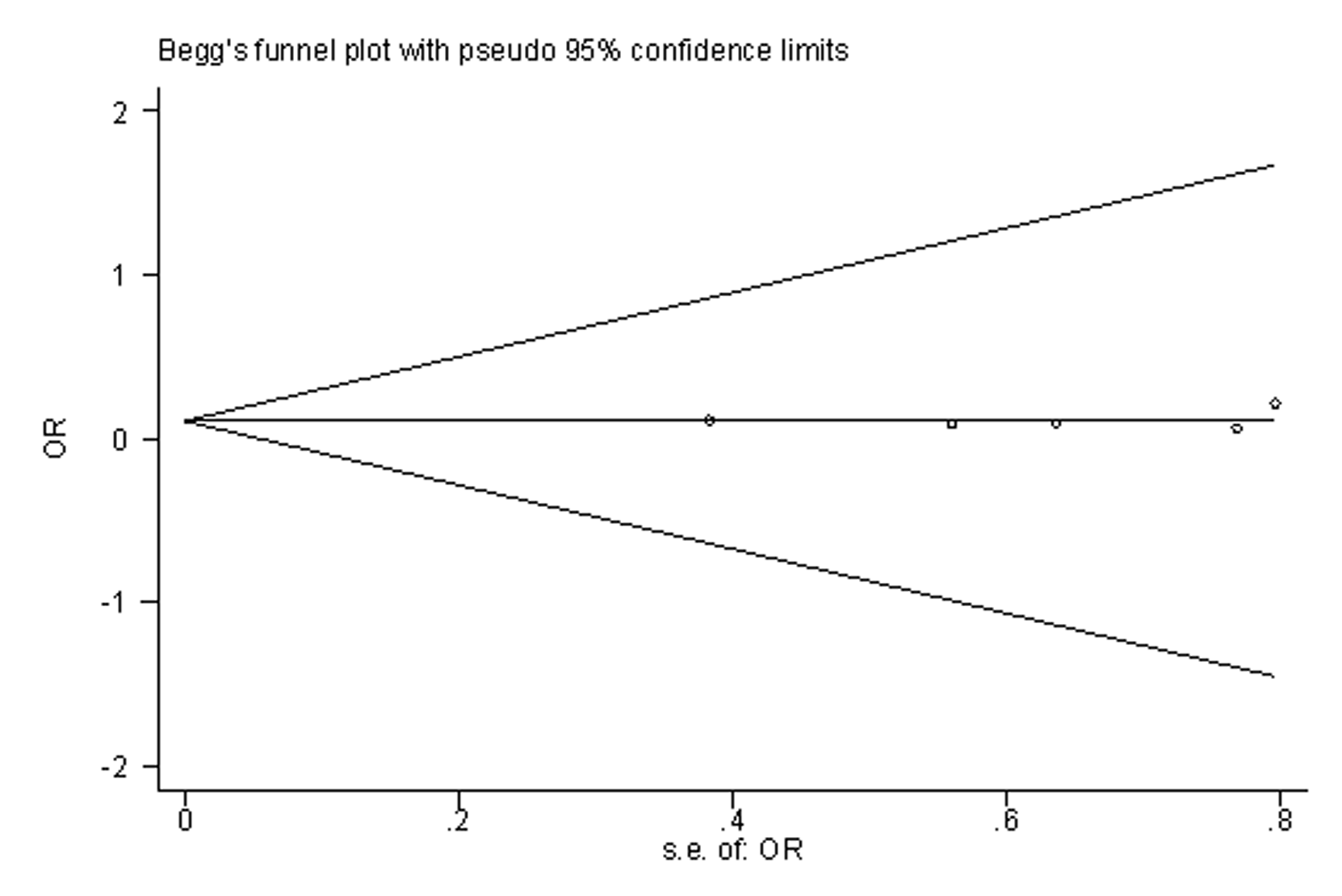


D


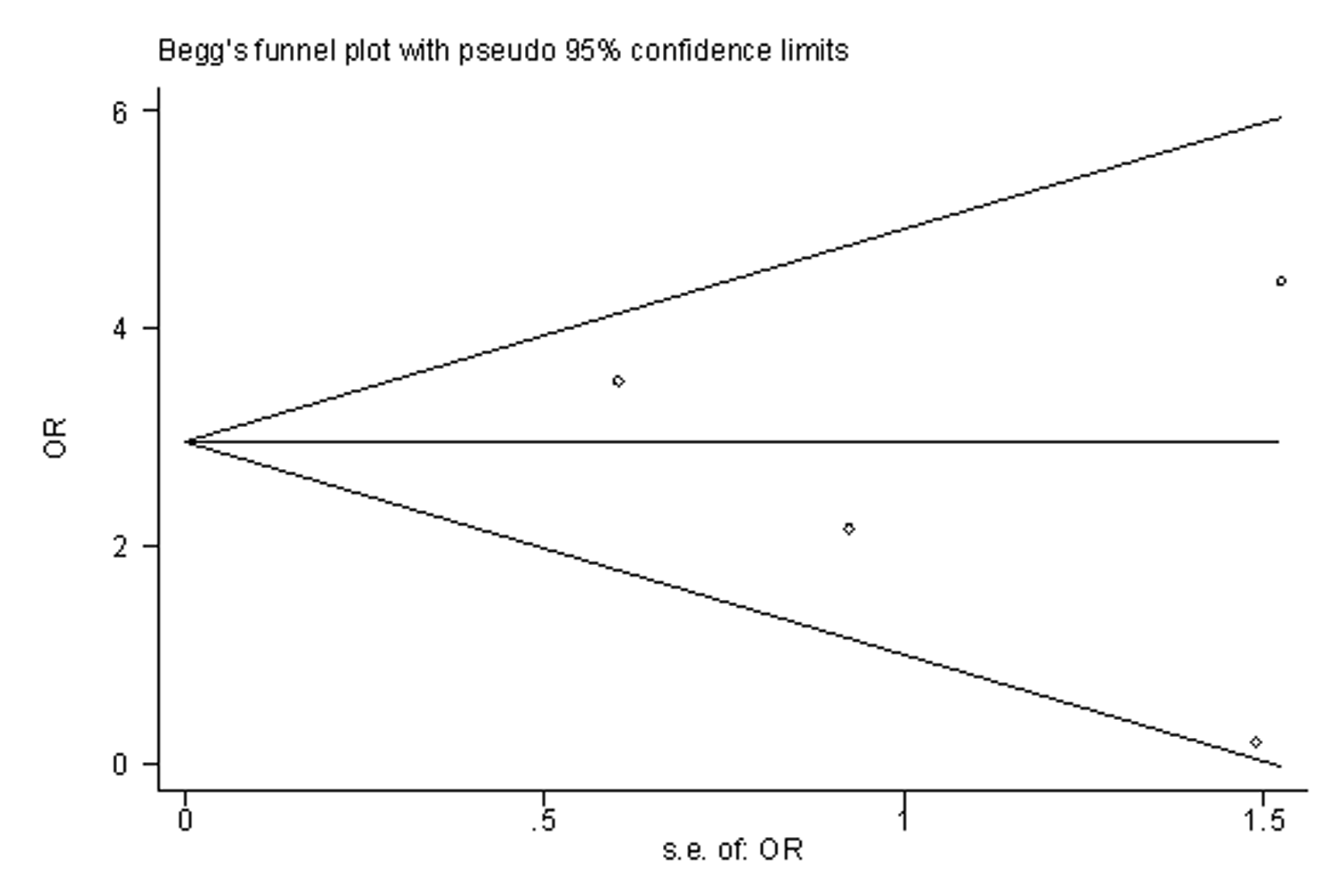


E


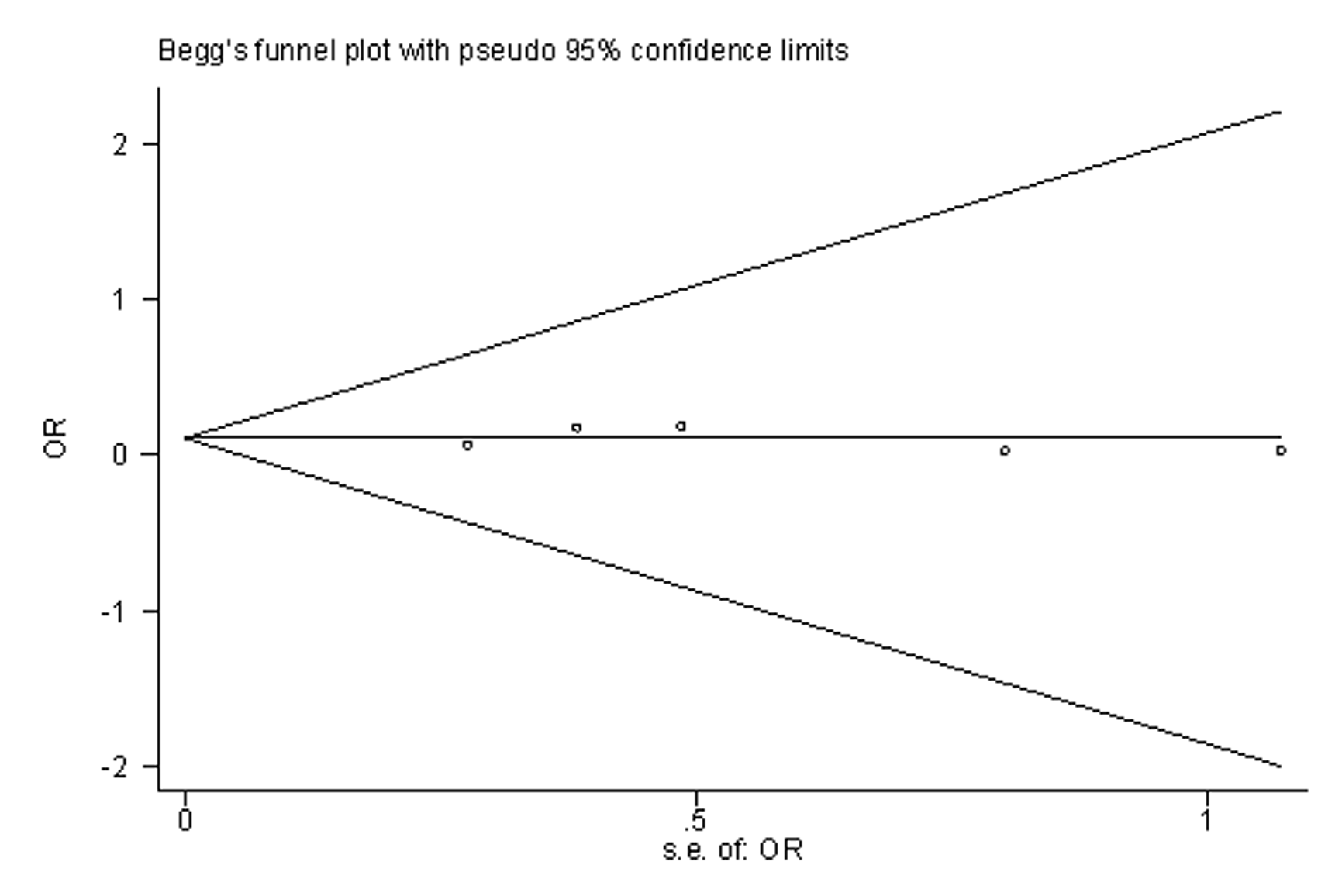


F

**Figure 1. Funnel plot for publication bias of the meta-analysis of HSCR and RET polymorphisms in dominant genetic model comparison. ( A: rs1800858; B: rs1800860.;C:rs1800861;D:rs10900297;E:rs26847582;F:rs2435357)**
