# Supplementary material for: RET and PHOX2B Genetic Polymorphisms and Hirschsprung's Disease Susceptibility: A Meta-Analysis
Source: PLoS One. 2014 Mar 20;9(3):e90091. doi: 10.1371/journal.pone.0090091 (PMC3961244; doi:10.1371/journal.pone.0090091)
Supplement: Supplement S4 — Sensitivity analysis after exclusion of three studies deviating from HWE on the association of rs1800858 and rs1800861 gene polymorphisms with HSCR risk. (DOC) [file pone.0090091.s004.doc]

| Gene polymorph-ism | Number of  studies | Comparison | Test of association | | |  | Test ofheterogeneity | | |  |  |
| --- | --- | --- | --- | --- | --- | --- | --- | --- | --- | --- | --- |
|  |  |  | OR | 95%CI | *P* value |  | Q | *P* value | I^2^(%) |  |  |
| rs1800858 | 6 | AA vs GG | 8.56 | 2.47-29.71 | 0.001 |  | 68.14 | 0.000 | 92.7 |  |  |
|  | 6 | AA+GA vs GG | 3.58 | 1.46-8.81 | 0.005 |  | 54.78 | 0.000 | 90.9 |  |  |
|  | 6 | AA vs GA+GG | 5.91 | 2.55-13.69 | 0.000 |  | 5221 | 0.000 | 904 |  |  |
|  | 6 | A vs G | 3.46 | 1.73-6.95 | 0.000 |  | 94.79 | 0.000 | 94.7 |  |  |
| rs1800861 | 5 | GG vs TT | 7.41 | 4.89-11.23 | 0.000 |  | 7.77 | 0.100 | 48.5 |  |  |
|  | 5 | GG+TG vs TT | 3.10 | 2.11-4.57 | 0.000 |  | 5.22 | 0.265 | 23.4 |  |  |
|  | 5 | GG vs TT+TG | 6.15 | 3.87-9.76 | 0.000 |  | 1070 | 0.030 | 62.6 |  |  |
|  | 5 | G vs T | 3.42 | 2.38-4.91 | 0.000 |  | 12.91 | 0.012 | 69.0 |  |  |
|  |  |  |  |  |  |  |  |  |  |  |  |
|  |  |  |  |  |  |  |  |  |  |  |  |
|  | | | | | | | | | | | |
|  |  |  |  |  |  |  |  |  |  |  |  |
|  |  |  |  |  |  |  |  |  |  |  |  |

**Table 1 Sensitivity analysis after exclusion of studies deviating from HWE on the association of rs1800858 and rs1800861 gene polymorphisms with HSCR risk**
